# Unraveling the Origin of Photocatalytic Deactivation in CeO_2_/Nb_2_O_5_ Heterostructure Systems during Methanol Oxidation: Insight into the Role of Cerium Species

**DOI:** 10.1021/acs.jpcc.1c02812

**Published:** 2021-06-02

**Authors:** Lukasz Wolski, Oleg I. Lebedev, Colin P. Harmer, Kirill Kovnir, Hanen Abdelli, Tomasz Grzyb, Marco Daturi, Mohamad El-Roz

**Affiliations:** † Faculty of Chemistry, Adam Mickiewicz University, Poznań, Uniwersytetu Poznańskiego 8, Poznań 61-614, Poland; ‡ Normandie Univ, ENSICAEN, UNICAEN, CNRS, Laboratoire Catalyse et Spectrochimie, Caen 14050, France; § Normandie Univ, ENSICAEN, UNICAEN, CNRS, Laboratoire CRISMAT, Caen 14050, France; ∥ Department of Chemistry, Iowa State University, Ames, Iowa 50011, United States; ⊥ U.S. Department of Energy, Ames Laboratory, Ames, Iowa 50011, United States; # Department of Rare Earths, Faculty of Chemistry, Adam Mickiewicz University, Poznań, Uniwersytetu Poznańskiego 8, 61-614 Poznań, Poland

## Abstract

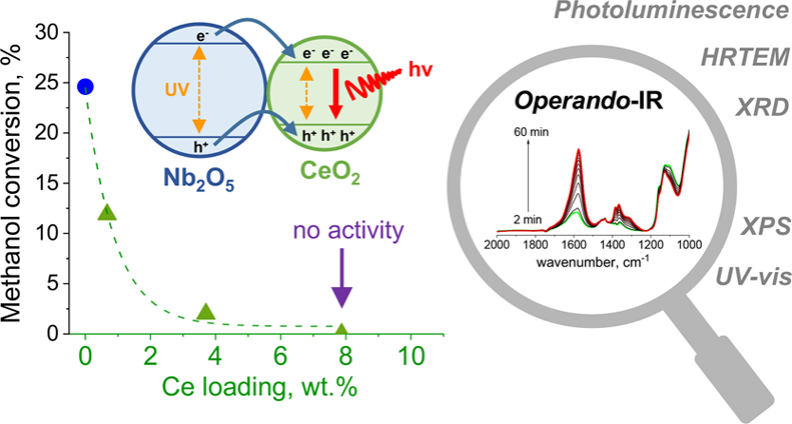

The study provides deep insight into the origin of photocatalytic deactivation of Nb_2_O_5_ after modification with ceria. Of particular interest was to fully understand the role of ceria species in diminishing the photocatalytic performance of CeO_2_/Nb_2_O_5_ heterostructures. For this purpose, ceria was loaded on niobia surfaces by wet impregnation. The as-prepared materials were characterized by powder X-ray diffraction, nitrogen physisorption, UV–visible spectroscopy, X-ray photoelectron spectroscopy, high-resolution transmission electron microscopy, and photoluminescence measurements. Photocatalytic activity of parent metal oxides (i.e., Nb_2_O_5_ and CeO_2_) and as-prepared CeO_2_/Nb_2_O_5_ heterostructures with different ceria loadings were tested in methanol photooxidation, a model gas-phase reaction. Deep insight into the photocatalytic process provided by *operando*-IR techniques combined with results of photoluminescence studies revealed that deactivation of CeO_2_/Nb_2_O_5_ heterostructures resulted from increased recombination of photo-excited electrons and holes. The main factor contributing to more efficient recombination of the charge carriers in the heterostructures was the ultrafine size of the ceria species. The presence of such highly dispersed ceria species on the niobia surface provided a strong interface between these two semiconductors, enabling efficient charge transfer from Nb_2_O_5_ to CeO_2_. However, the ceria species supported on niobia exhibited a high defect site concentration, which acted as highly active recombination centers for the photo-induced charge carriers.

## 1 Introduction

Cerium dioxide is one of the most intensively studied model heterogeneous catalysts. Hitherto, it has been established that ceria can be successfully used not only as a support for various active phases but it can also be involved as a promising active component for various reactions.^1^ Recently, cerium dioxide has been thoroughly studied for applications in advanced oxidation processes, e.g., photocatalytic oxidation^2−4^ or Fenton-like reactions.^5−7^ Many authors have reported that loading of ceria on the surface of semiconducting metal oxides is an efficient method to improve the photocatalytic performance of parent oxides. For instance, Zhu et al.^8^ have documented that CeO_2_/ZnO composites displayed highly enhanced photocatalytic activity in Rhodamine B (RhB) degradation compared to pristine ZnO and CeO_2_. The increase in activity of the CeO_2_/ZnO heterostructures was attributed by the authors to formation of Z-scheme heterojunction, which improved the separation of photo-generated charge carriers. Improved efficiency of charge carrier separation resulting from formation of heterojunctions has also been reported for CeO_2_/TiO_2_ systems in photooxidation of toluene^9^ and photodegradation of phenazopyridine drugs.^10^ However, the above-mentioned paradigm concerning the positive role ceria modifier plays in improving the photocatalytic activity of various semiconducting metal oxides has been recently rebutted by Morlando et al.^11^ The authors found that deposition of CeO_2_ nanodots on the surface of TiO_2_ led to a significant decrease in photocatalytic activity of the as-formed composite materials. The authors claimed that the decrease in activity of CeO_2_/TiO_2_ nanocomposites may have resulted from scavenging of reactive oxygen species by ceria species, increased recombination of photo-excited charge carriers caused by ceria doping and/or UV shielding effects from ceria loading on the surface of TiO_2_. Deactivation of photocatalysts after loading of ceria was also observed for CeO_2_/ZnO heterostructures.^12^ According to the authors, deactivation of this catalytic system arose from the presence of ultrafine CeO_2_ nanoparticles with existing surface defects, which could impart some form of reactive oxygen species scavenging property. However, no evidence supporting the above-mentioned hypotheses, explaining deactivation of TiO_2_ or ZnO photocatalysts after loading of ceria species, has been provided.

Besides ZnO and TiO_2_, niobium pentoxide (Nb_2_O_5_) is another semiconductor, which has been successfully used in photocatalytic reactions.^13−17^ Niobia is known for its high Brønsted acidity, which can play an important role in controlling the selectivity of various processes.^14,18,19^ Previous reports show that the photocatalytic activity of Nb_2_O_5_ can also be improved by forming heterojunctions with other semiconductors (e.g., Nb_2_O_5_/TiO_2_,^20,21^ Nb_2_O_5_/ZnO,^22^ Nb_2_O_5_/Bi_2_WO_6_,^23^ NiO/Nb_2_O_5_,^24^ and Nb_2_O_5_/g-C_3_N_4_^25^). However, studies concerning the influence of ceria modifier on the photocatalytic activity of niobium pentoxide are sparse. Ferraz et al.^26^ have tested CeO_2_/Nb_2_O_5_ heterostructures with low ceria loadings (up to 2 wt % of CeO_2_ in the composite) in photocatalytic degradation of phenol and methylene blue under UV light (λ = 254 nm). The photocatalysts had a low surface area of ca. 15 m^2^/g and consisted of relatively large ceria particles (ca. 14 nm in diameter) supported on Nb_2_O_5_. The authors have established that a loading of 0.3 wt % of CeO_2_ on the surface of niobia improved photocatalytic performance of the as-formed heterostructure, but at a higher ceria loading in the composite, the activity was slightly lower than that observed for parent Nb_2_O_5_. Thus, no remarkable deactivation effect was reported by the authors after modification of niobia with ceria. It is worth noting that the experiments were performed under monochromatic UV light (λ = 254 nm), which can activate both methylene blue and phenol. It means that both photocatalytic degradation and photochemical degradation processes could occur. Thus, the above-mentioned photocatalytic processes in the liquid phase are very complex and do not allow to gain clear information about the role of ceria modifier in controlling the photocatalytic performance of niobia-based heterostructures. In view of the recent results, reporting the ambiguous negative/positive role of ceria species in CeO_2_/ZnO and CeO_2_/TiO_2_ heterostructures, a fundamental evaluation of the ceria species’ role in controlling the activity of niobia-based photocatalysts, is crucial for rational development of ceria-containing photocatalysts.

The present study establishes the influence of ceria modifier on the structure, texture, and photocatalytic performance of niobium pentoxide. Ceria species were loaded on the niobia surface by wet impregnation. Photocatalytic activity of parent metal oxides (i.e., Nb_2_O_5_ and CeO_2_) and as-prepared CeO_2_/Nb_2_O_5_ heterostructures with different ceria loadings was tested in methanol oxidation, a well-known model gas-phase reaction that allows us to determine the relationship between properties of materials and their catalytic performance. *Operando-*IR techniques provided insight into the mechanism of the photocatalytic process across CeO_2_/Nb_2_O_5_ heterostructures while elucidating the role individual components of each heterostructure play in methanol photooxidation.^27−29^

## 2 Experimental Section

### 2.1 Synthesis of Nb_2_O_5_

Niobium pentoxide was synthesized using the hydrothermal procedure described by Murayama et al.^30^ In a typical synthesis route, ammonium niobate(V) oxalate hydrate (Sigma-Aldrich, C_4_H_4_NNbO_9_·H_2_O, 99.99%) (9.0894 g, 30 mmol) was dissolved in 200 mL of deionized water. Following 1 h of vigorous stirring, the pellucid solution was sealed in a Teflon-lined stainless steel autoclave and heated for 24 h at 175 °C. The solid formed during hydrothermal treatment was then separated by filtration, washed with deionized water, dried at room temperature, and calcined for 2 h at 400 °C (temperature ramp: 1.6 °C/min). The as-prepared material was denoted as Nb_2_O_5_.

### 2.2 Loading of Ceria Species on the Niobia Support

Ceria species were loaded on the surface of Nb_2_O_5_ via a facile wet impregnation method. In a typical synthesis, 6 g of as-prepared Nb_2_O_5_ was dispersed in 10 mL of deionized water. Meanwhile, cerium(III) nitrate hexahydrate (Sigma-Aldrich, 99.99%) was dissolved in 35 mL of deionized water (the amount of cerium source was adjusted to obtain 1.0, 5.0, or 10.0 wt % of Ce on the niobia support). Next, the solution of cerium(III) nitrate hexahydrate was stirred into the mixture containing niobium pentoxide dispersed in water. Following 1 h of agitation at room temperature, the mixture was transferred to a round-bottom flask and sonicated for 10 min. In the next step, water was evaporated from the mixture using a rotary evaporator and the as-obtained powder was dried in a furnace at 80 °C for 12 h. Finally, the dry powder was calcined at 400 °C for 2 h (temperature ramp: 1.6 °C/min). The as-prepared ceria-modified catalysts containing 1.0, 5.0, or 10.0 wt % of Ce were labeled as Ce1/Nb_2_O_5_, Ce5/Nb_2_O_5_, and Ce10/Nb_2_O_5_, respectively.

Mechanical CeO_2_/Nb_2_O_5_ mixtures were prepared using Nb_2_O_5_ and commercial CeO_2_. For this purpose, appropriate amounts of metal oxides were mixed with a small amount of deionized water and then crushed in an agate mortar to get a homogeneous mixture. Next, the as-obtained mechanical mixtures of metal oxides were dried overnight at 70 °C to obtain dry powders. The as-prepared materials were used as reference samples.

### 2.3 Characterization of Catalysts

The catalysts were characterized with the use of inductively coupled plasma-optical emission spectrometry (ICP-OES), nitrogen physisorption, powder X-ray diffraction (PXRD), high-resolution transmission electron microscopy (HRTEM), UV–visible spectroscopy (UV–vis), X-ray photoelectron spectroscopy (XPS), and photoluminescence measurements (PL).

Powder X-ray diffraction patterns were collected on a benchtop Rigaku Miniflex 600 with Cu K_α_ radiation (λ = 1.5406 Å) and Ni-K_β_ filter. Peak locations of each sample were refined against a Si internal standard (Si 640d, *Fd*3*m*, *a* = 5.43123 Å) via Rietveld refinement in GSAS-II.^31^ Rietveld refinement was initiated by determining sample displacement from the Si internal standard. Then, size and preferred orientation were refined to best fit each sample before refining unit cell parameters.

Transmission electron microscopy (TEM) was performed using a JEM ARM200F cold FEG probe and image aberration corrected microscope, operated at 200 kV and equipped with a large angle CENTURIO EDX detector, Orius Gatan CCD camera, and Quantum GIF. The TEM samples were prepared in conventional way—depositing a solution of the material in ethanol on a carbon holey Cu grid.

Diffuse reflectance UV–vis spectra (DR UV–vis) were recorded on a Varian Cary 300 Scan spectrophotometer equipped with a diffuse reflectance accessory. Spectra were recorded at room temperature from 200 to 800 nm using Spectralon as a reference material.

Photoluminescence properties were studied at room temperature using a PIXIS:256E Digital CCD camera equipped with an SP-2156 Imaging Spectrograph (Princeton Instruments) and Opolette 355LD UVDM tunable laser as the excitation source (with a repetition rate of 20 Hz; 0.5 mJ pulse energy at 250 nm). All spectra were corrected for spectral response of the equipment. The beam size and laser powers were determined by a 10A-PPS power meter (Ophir Photonics).

The N_2_ adsorption–desorption isotherms were obtained at −196 °C using a Micromeritics ASAP 2020 Physisorption Analyzer. Before the measurements, samples were degassed at 120 °C for 10 h. The surface area of the materials obtained was estimated by the Brunauer–Emmett–Teller (BET) method.

X-ray photoelectron spectroscopy (XPS) was performed using an ultra-high vacuum photoelectron spectrometer based on a Phoibos150 NAP analyzer (Specs, Germany). The analysis chamber was operated under vacuum with a pressure close to 5 × 10^–9^ mbar, and the sample was irradiated with a monochromatic Al Kα (1486.6 eV) radiation. Any charging that occurred during the measurements (due to incomplete neutralization of ejected surface electrons) was accounted for by rigidly shifting the entire spectrum by a distance needed to set the binding energy of the C1s assigned to adventitious carbon to the assumed value of 284.8 eV.

### 2.4 Photocatalytic Tests

The photocatalysts were pressed into self-supported wafers of similar density (Ø = 16 mm, *m* ≈ 11.7 mg/cm^2^) and about 65 ± 2 μm in thickness. Thus, FTIR spectra of different samples recorded in transmission mode could be directly and quantitatively compared without any additional normalization. The outlet gas phase evolution was followed by both IR spectroscopy and mass spectrometry. FTIR spectra of the outlet gas phase and the samples were collected with a Nicolet 5700 FTIR spectrometer (64 scans/spectrum) equipped with an MCT detector. The *operando*-IR system was connected to a flow setup.^32^ Gases were introduced into the lines by mass flow controllers. The system allows the two gas mixtures, the so-called “activation” and “reaction” flows, to be prepared and sent independently to the reactor cell. The “sandwich” type reactor cell used in this study is described in ref (33). It was made of a stainless steel cylinder that carries a toroidal sample holder in its center, where the catalyst self-supporting wafer was placed. Tightness was obtained by O-rings, and the dead volume (typically defined as the residual space between each sample face and the windows) was reduced to about 0.4 mL by filling the empty space with KBr windows placed on each side of the sample holder. The sample analysis was made possible without the superposition of the gas phase signal and fluid dynamics. Gases were introduced to the sample and evacuated by two 1/8 inch OD pipes connected to the opposite sides of the sample holder. In this study, UV irradiation was carried using a UV light guide (A10014-50-0110) mounted at the entrance to the IR cell and connected to a polychromatic light of Xe-Hg lamp (LC8 spot light Hamamatsu, L10852, 200 W) equipped with a filter to enable monochromatic UV irradiation (λ = 365 nm). More details on the *operando*-IR system for photocatalysis can be found in refs (33) and (34). The employed configuration allowed a low partial pressure of methanol to be achieved using a saturator at a controlled temperature. The gas mixture composition was fixed then at 0.12 vol. % methanol and 20 vol. % O_2_ in Ar, and the total flow was adjusted to 20 cm^3^/min. Outlet gases were characterized by a Pfeiffer Omnistar mass spectrometer. FTIR spectra of the gas phase were collected using a gas microcell. The conversions were calculated at the steady state.

## 3 Results and Discussion

### 3.1 Photocatalytic Tests

**Figure 1:**
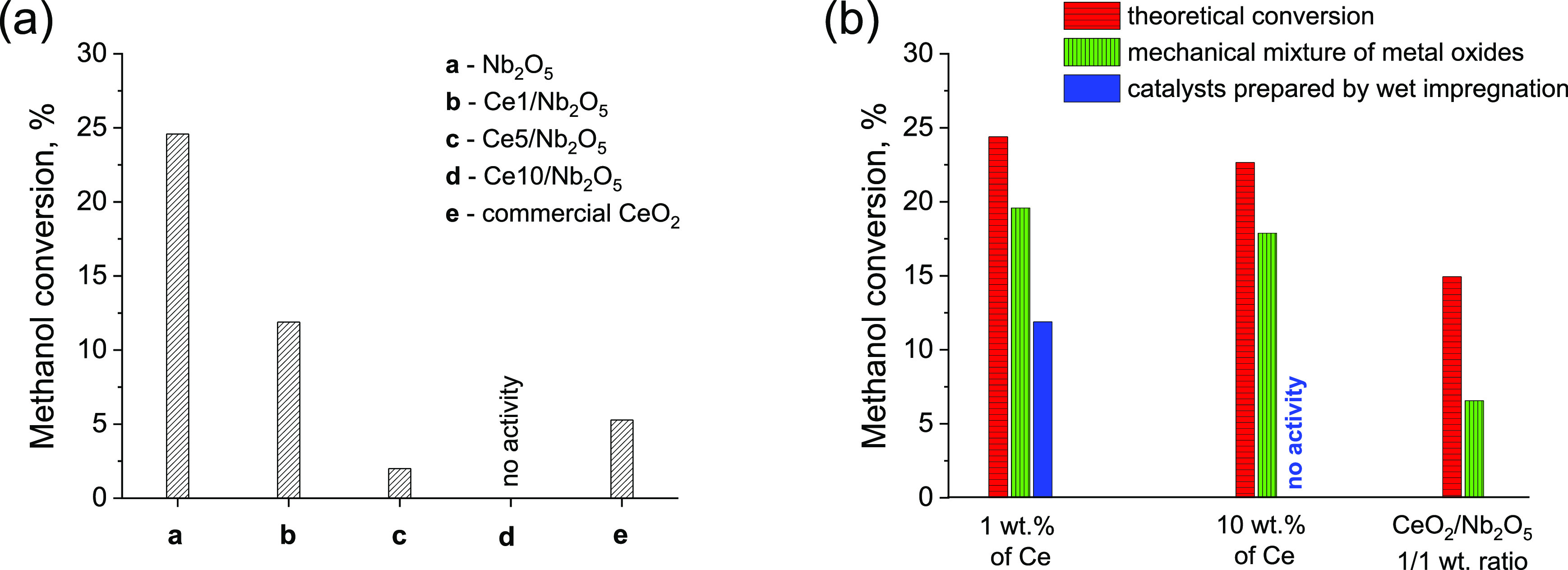
(a) Activity of catalysts in methanol photooxidation at the steady state for 2 h of reaction. (b) Graph presenting differences between activity of ceria-modified catalysts prepared by wet impregnation, mechanical mixtures of metal oxides, and theoretical methanol conversion expected at the given concentration of Nb_2_O_5_ and CeO_2_ in the composite materials (theoretical conversion of methanol was estimated by summing up activity of given amounts of commercial CeO_2_ and parent Nb_2_O_5_ in the heterostructures). A mechanical mixture of metal oxides was prepared using parent Nb_2_O_5_ and commercial CeO_2_ (see Section 2).

Photocatalytic activity of materials was tested via methanol oxidation in the gas phase. The results are shown in Figure 1a. The highest methanol conversion of 24.6% was observed for parent Nb_2_O_5_. Commercial CeO_2_ was significantly less active than pristine niobium pentoxide and its activity at 5.3%. As can be seen from Figure 1a, all ceria-modified samples exhibited significantly lower activity than parent Nb_2_O_5_. In the case of Ce1/Nb_2_O_5_, activity was reduced by more than 50% compared to pristine niobia (24.6% vs 11.9% of methanol conversion for Nb_2_O_5_ and Ce1/Nb_2_O_5_, respectively). A more pronounced decrease in the activity of the composite catalysts was observed for Ce5/Nb_2_O_5_. Interestingly, for Ce10/Nb_2_O_5_, photocatalytic activity of the heterostructure was totally quenched. There were no products formed during the methanol photooxidation over this catalyst.

To gain deeper insight into the quenching of catalysts’ activity after loading of ceria on niobia, we performed additional measurements with mechanical mixtures of metal oxides as reference samples. We found that activity of these mechanical mixtures of metal oxides containing 1 wt % and 10 wt % of Ce was only slightly lower than that observed for unmodified Nb_2_O_5_ (see Figure 1b). Interestingly, the activity of mechanical mixtures of CeO_2_ and Nb_2_O_5_ oxides was not quenched even at a very high concentration of CeO_2_ in the composite material (Nb_2_O_5_:CeO_2_ weight ratio of 1:1). As can be seen from Figure 1, such a mixture of metal oxides was still slightly more active than commercial CeO_2_. To shed light on the role of ceria species in deactivation of ceria-modified samples, prepared by wet impregnation, we compared the activity of these materials with the activity of mechanical mixtures of metal oxides and theoretical activity of the composite materials expected at a given concentration of CeO_2_ and Nb_2_O_5_ in the heterostructures (theoretical methanol conversion was estimated by summing up the activity of a given amount of Nb_2_O_5_ and commercial CeO_2_ in the composite). According to our results, the activity of mechanical mixtures of metal oxides was always lower than the expected theoretical values but was still significantly higher than the activity of samples prepared by wet impregnation (see Figure 1b). This observation led us to conclude that deactivation of the composite catalysts prepared by wet impregnation should be somehow related to the unique properties of these materials and their interaction.

**Figure 2:**
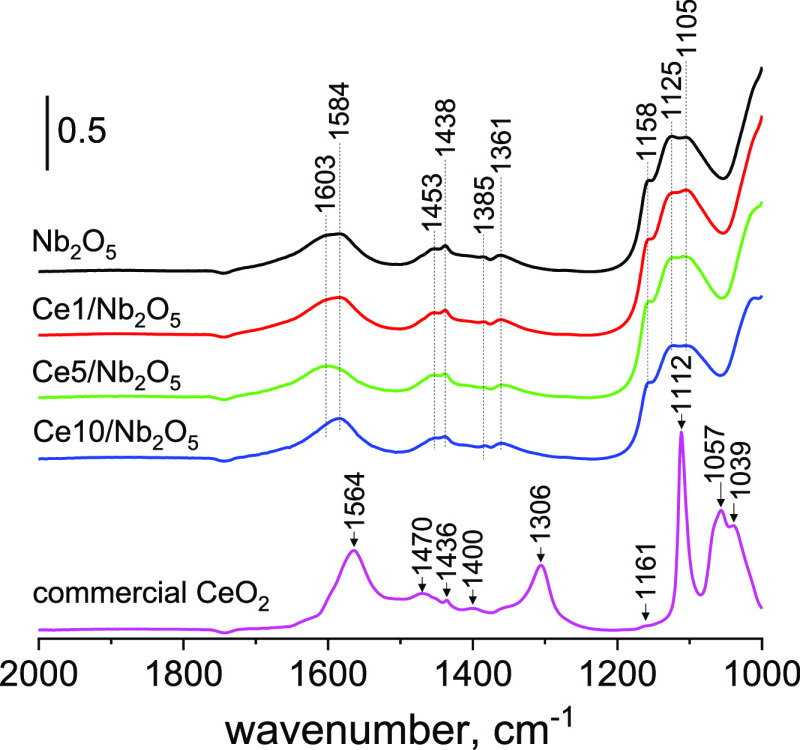
FTIR spectra of catalyst surfaces at the steady state during methanol adsorption under dark conditions.

To further probe the role of the ceria modifier in the photocatalytic process, *operando*-IR studies have been performed. According to the literature,^35^ the first step of methanol photooxidation is the adsorption of methyl alcohol on the catalyst surface and consequently the formation of surface methoxy species. *Operando*-IR studies show that exposure of all catalysts to the gas feed led to immediate disappearance of IR bands typical of surface hydroxyl groups of niobia and ceria (e.g., IR bands at ca. 3662 cm^–1^ characteristic of bridged OH groups in the fluorite structure of ceria^36,37^) and appearance of several new IR bands typical of adsorbed methanol molecules (e.g., IR bands in the range from 2750 to 3000 cm^–1^;^34,38^ see Figure S1, Supporting Information). Detailed analysis of the adsorbed methoxy species, in the range of wavelengths shown in Figure S1, is problematic since the typical C–H vibration bands overlap with the characteristic O–H vibration bands of adsorbed water. Thus, detailed analysis of adsorbed species on catalyst surfaces was performed on the basis of FTIR spectra in the range of wavenumbers where characteristic vibrations of adsorbed methoxy groups are not overlapped with other vibrational bands (i.e., at 1200–1000 cm^–1^^39^). Figure 2 shows surface FTIR spectra of catalysts at the equilibrium state during methanol adsorption under dark conditions. In the case of ceria, it is easy to identify the bands due to linearly (1112 cm^–1^) and two-fold (1057 cm^–1^) coordinated methoxy species on Ce^4+^.^39^ The components at 1039 cm^–1^ is assigned to bridged species on Ce^4+^ cations in the proximity of an oxygen vacancy.^39^ The other bands observed at higher wavenumbers are due to carbonate impurities belonging to ceria exposure to air^39^ and to residual oxalates on the surface of niobia. Analysis of IR spectra recorded for niobia-based samples allowed discrimination of three components at ca. 1158, 1125, and 1105 cm^–1^. The first component at ca. 1158 cm^–1^ is characteristic of ρ(CH_3_) rocking mode of methoxy species,^40^ while the latter two IR bands are typical of ν(OC) vibration modes of linearly (1125 cm^–1^) and may be two-fold (or another linear species coordinated on different exposed crystal planes, at 1105 cm^–1^) coordinated methoxy species on niobia.^41^ We found no significant differences in the forms and concentration of methoxy species on the surface of unmodified Nb_2_O_5_ and ceria-modified samples prepared by wet impregnation. Even at a relatively high loading of ceria in Ce10/Nb_2_O_5_ catalyst, no noticeable IR bands typical of aforementioned methoxy species bonded to the ceria surface were found. In view of these observations, we concluded that quenching of niobia activity through loading of ceria by wet impregnation cannot be attributed to covering of the niobia surface by ceria species and/or hindering of niobia ability to adsorb methanol molecules.

**Figure 3:**
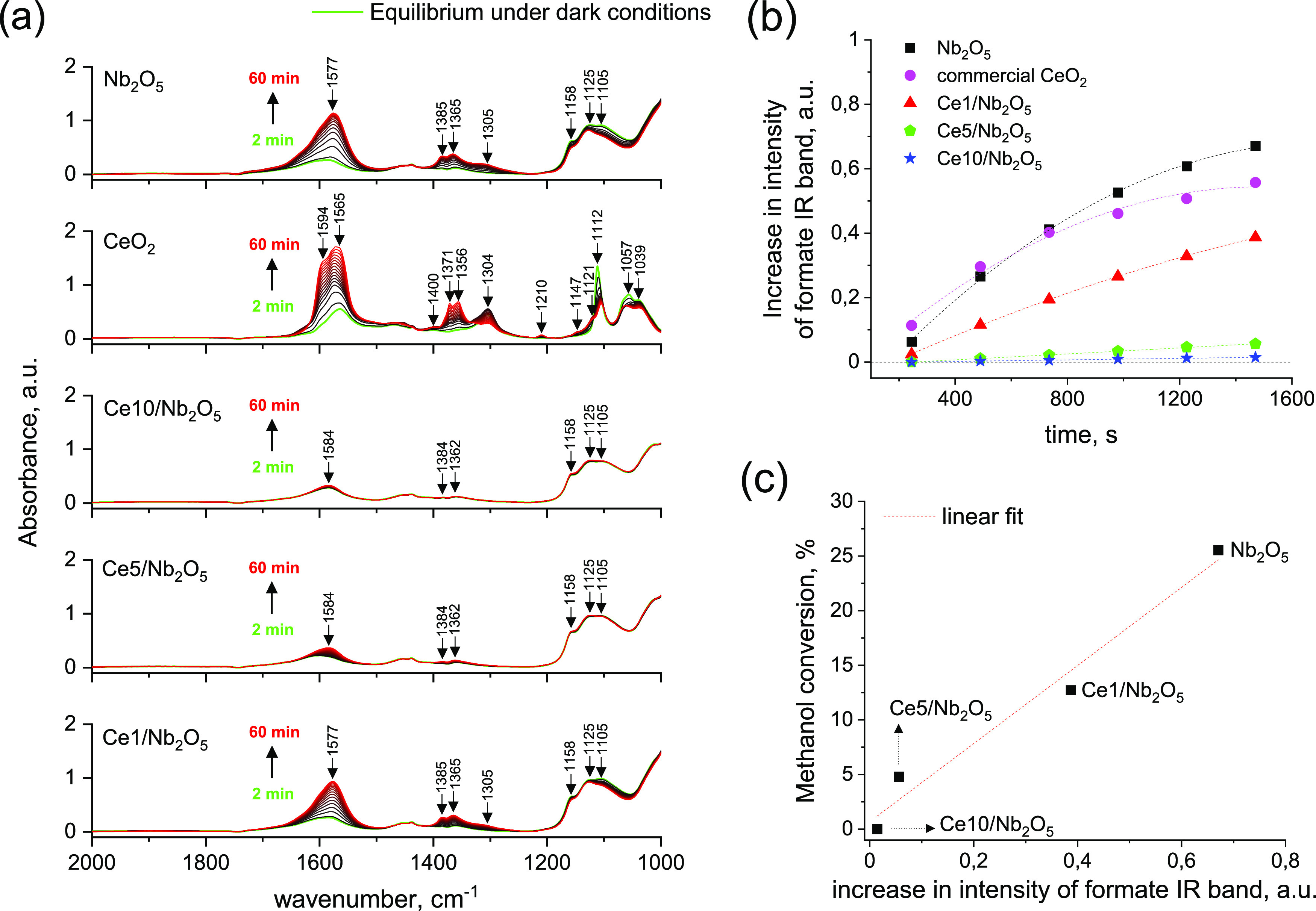
(a) FTIR spectra of catalyst surfaces recorded at the beginning of photocatalytic oxidation of methanol under UV light (λ = 365 nm). (b) Graph presenting changes in intensity of the most intense IR band typical for adsorbed formate species (i.e., band at 1577 cm^–1^ for Nb_2_O_5_ and Ce1/Nb_2_O_5_; band at 1584 cm^–1^ for Ce5/Nb_2_O_5_ and Ce10/Nb_2_O_5_; and band at 1565 cm^–1^ for commercial CeO_2_) at the beginning of photocatalytic process. (c) Relationship between the activity of catalysts and increase in intensity of the most intense IR band typical of adsorbed formate species. The variations of the IR band’s intensities (in panels (b) and (c)) were measured by the dedicated tool in OMNIC software, after having subtracted the IR spectrum of the catalyst at the equilibrium steady state during methanol adsorption under dark conditions, from the IR spectrum after about 25 min of photocatalytic reaction.

The second step in photocatalytic oxidation of methanol is oxidation of adsorbed methoxy species to formaldehyde.^35^ The as-formed formaldehyde can then be desorbed from the catalyst surface or further transformed into other products such as methyl formate, formic acid, or carbon dioxide. According to the literature, oxidation of formaldehyde to other products leads to appearance of some reaction intermediates, such as adsorbed formate species, characterized by the vibrational bands at ca. 1565, 1371, and 1356 cm^–1^ for commercial CeO_2_ and ca. 1577, 1385, and 1365 cm^–1^ for niobia-based photocatalysts.^33,35^ As can be seen from sample FTIR spectra of pristine Nb_2_O_5_ and commercial CeO_2_ shown in Figure 3a, typical IR bands for these reaction intermediates showed up immediately after irradiation of the catalysts with UV light and continued to increase the intensity upon irradiation. The increase in intensity of typical IR bands of adsorbed formate species was associated with a decrease in intensity of characteristic IR bands of adsorbed methanol molecules (IR bands in the range of 1200–1000 cm^–1^). This observation led us to conclude that methanol is efficiently oxidized on the surface of these two unmodified metal oxides. Interestingly, it was revealed that methanol oxidation on ceria proceeds until formation of CO_2_, as witnessed by the formation of bidentate carbonates on the surface of the samples (bands at 1594 and 1304 cm^–1^).^39^ However, a different trend was observed for ceria-modified samples prepared by wet impregnation. As can be seen from Figure 3b, the changes in intensity of IR bands characteristic of adsorbed formate species was significantly suppressed for the Ce1/Nb_2_O_5_ and Ce5/Nb_2_O_5_ catalysts. In the case of a reaction with the use of Ce10/Nb_2_O_5_, almost no changes in intensity of IR bands typical of adsorbed formate species were found. Also, no changes were observed for this catalyst in the range of wavenumbers characteristic of adsorbed methoxy species (see Figure 3a). It is worth noting that for all niobia-based samples methanol conversion was found to be proportional to the intensity of IR bands characteristic of adsorbed formate species (see Figure 3c). Thus, IR experimental data indicated that a decrease in activity of heterostructures prepared by wet impregnation resulted from their reduced ability to oxidize methanol molecules adsorbed on the niobia surface.

### 3.2 Characterization of Catalysts

To understand the origin of photocatalytic deactivation of ceria-modified samples prepared by wet impregnation, the as-prepared catalysts were precisely characterized by a variety of complementary methods, providing information about their composition, structure, texture, optical, and electronic properties.

To confirm the presence of ceria species on the niobia surface, the chemical composition of catalysts was analyzed with the use of ICP-OES. As can be seen from Table 1, for all ceria-containing catalysts, the real loading of Ce was only slightly lower than the assumed values and was found to be 0.7, 3.7, and 7.9 wt % of Ce for Ce1/Nb_2_O_5_, Ce5/Nb_2_O_5_, and Ce10/Nb_2_O_5_, respectively.

**Table 1:** Characteristics of Niobia-Based Catalysts

catalyst	BET surface area [m^2^/g]	average pore size^a^ [nm]	Ce loading^b^ [wt %]
Nb_2_O_5_	157	8.8	
Ce1/Nb_2_O_5_	153	9.0	0.7
Ce5/Nb_2_O_5_	156	8.3	3.7
Ce10/Nb_2_O_5_	143	8.1	7.9
CeO_2_^c^			

a Estimated with the use of the BJH method from the adsorption branch.

b Determined with the use of ICP-OES.

c Commercial CeO_2_.

**Figure 4:**
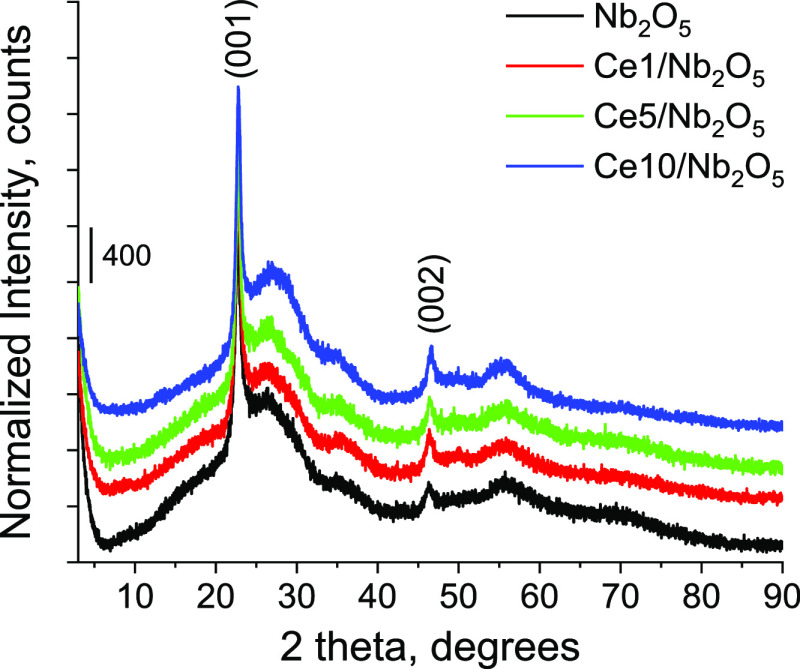
PXRD patterns of catalysts normalized to the intensity of the (001) peak.

Structure of materials was characterized by powder X-ray diffraction (λ = 1.5406 Å). As can be seen from Figure 4, PXRD patterns of all samples exhibited two distinct diffraction peaks at 22.7 and 46.3° 2θ, which are similar to the reported PXRD pattern of the deformed orthorhombic Nb_2_O_5_ phase.^30^ It should be noted that no crystalline CeO_2_ or other cerium species was identified for all ceria-modified materials, indicating that ceria species existed in amorphous form or were highly dispersed on the niobia surface.

Detailed analysis of electron diffraction (ED) patterns and high-resolution TEM images of niobium pentoxide led us to observe discrepancy between the previously reported deformed orthorhombic structure of Nb_2_O_5_^30^ and the structure obtained in this study. For this reason, identification of a niobia structural model was determined by first attempting to fit the PXRD data to known structures. Although we did not find any matches to known structurally characterized Nb–O binaries, a search among tantalum oxides (note the similar size of Ta and Nb) revealed orthorhombic Ta_2_O_5_ crystallizing in the *Cmmm* space group as a reasonable fit.^42^ Ta_2_O_5_ is composed of Ta–O octahedra with Ta on the *ab* plane bridged by equatorial O atoms and stacked along the *c* axis by axially bridging O atoms. In this model, the equatorial O atoms are fully occupied with half occupancy for the axial O site. This is an average structure, which was shown to be incommensurately modulated. The equivalent atomic size and bonding characteristics between Nb and Ta allowed us to build an isostructural Nb_2_O_5_ model (Figure S2, Supporting Information). A further database search verified no previously reported Nb–O binaries in the orthorhombic *Cmmm* space group.

Analysis of PXRD data collected with the Si internal standard demonstrates that loading of ceria on niobia by wet impregnation resulted in a slight shift of the diffraction peaks at 22.7 and 46.3° 2θ toward higher angles (see Figure S3, Supporting Information). Rietveld refinement of our Nb_2_O_5_ model against PXRD data from each sample collected with a Si internal standard revealed a slight compression of the *c* parameter of the niobia structure with increasing Ce loading (see Table 2). The highest decrease in the *c* parameter value was typical of the sample with the highest concentration of ceria modifier, i.e., Ce10/Nb_2_O_5_. In view of these observations, we claim that ceria species loaded on Nb_2_O_5_ by wet impregnation strongly interacted with the niobia support.

**Table 2:** Calculated Unit Cell Parameter c Obtained from Rietveld Refinement of PXRD Data with the Si Internal Standard

catalyst	*c* parameter [Å]	Rwp^a^ [%]	GoF^b^
Nb_2_O_5_	3.9147(4)	2.84	1.22
Ce1/Nb_2_O_5_	3.9146(3)	2.88	1.24
Ce5/Nb_2_O_5_	3.9076(3)	3.2	1.39
Ce10/Nb_2_O_5_	3.9009(4)	3.56	1.66

a Weighted residual of the least-squares refinement.

b Goodness of fit.

**Figure 5:**
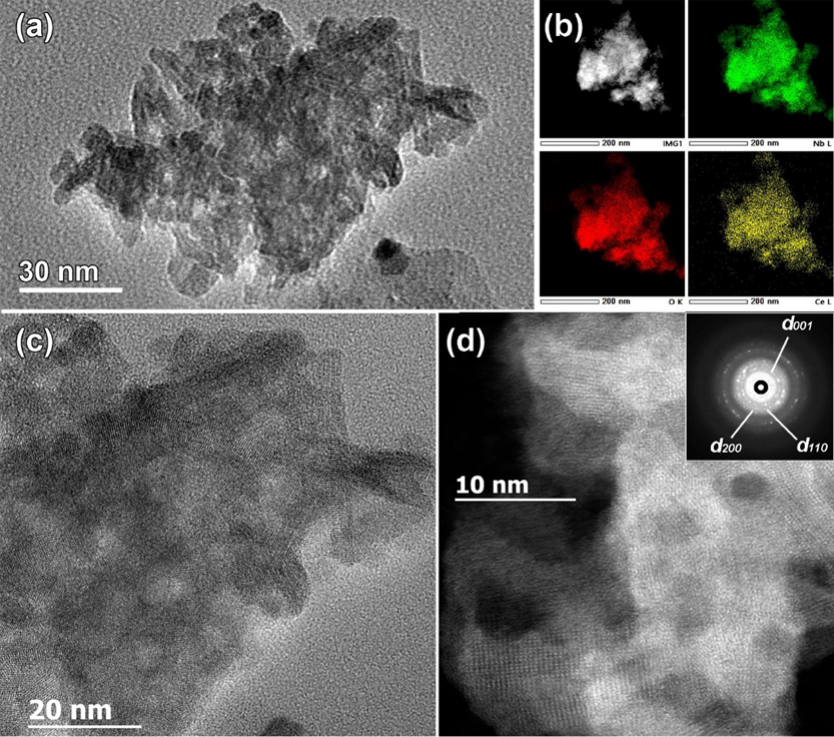
(a) Bright-field low magnification TEM image of the representative ceria-modified sample prepared by wet impregnation. (b) Low magnification HAADF-STEM image and simultaneously acquired EDX elemental mapping of Nb L, Ce L, and O K. (c) HRTEM and (d) high-resolution HAADF-STEM images of the representative Ce/Nb_2_O_5_ sample and corresponding ring ED pattern indexed based on the orthorhombic *Cmmm* Nb_2_O_5_ structure (*a* = 6.62 Å; *b* = 3.60 Å, *c* = 3.91 Å) obtained from PXRD.

To confirm the novel orthorhombic *Cmmm* structure of Nb_2_O_5_ proposed by PXRD, electron diffraction (ED), high-angle annular dark field scanning TEM (HAADF-STEM) imaging, and annular bright-field STEM (ABF-STEM) analyses were performed. The chemical composition and Ce distribution over the samples was confirmed by STEM-EDX elemental mapping. It was found that, irrespective of Ce amount, ceria-modified samples showed similar features as parent Nb_2_O_5_ (see Figure 5 and Figure S4, respectively). All samples consisted of needle-type nanostructures with typical dimensions: length of around 20–40 nm and diameter of 7–10 nm. They were stuck together in a random array, creating nanopores in the volume of the sample. The corresponding ring ED pattern of ceria-modified niobium pentoxide (Figure 5d, inset) can be fully indexed based on the proposed orthorhombic *Cmmm* structure. No extra rings belonging to Ce or Ce–O structures are present, in agreement with PXRD data. EDX elemental mapping in STEM mode evidenced homogeneous distribution of ceria species over the Nb_2_O_5_ sample (Figure 5b). STEM–EDX and ED studies support the conclusion drawn based on PXRD studies regarding the amorphous character or very high dispersion of ceria species on the niobia surface.

**Figure 6:**
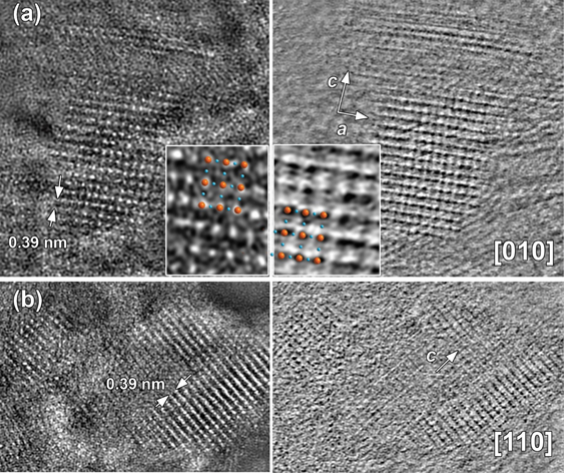
HAADF-STEM (left panel) and simultaneously acquired ABF-STEM (right panel) high-resolution images along the two main crystallographic zone axes, (a) [010] and (b) [110], of the orthorhombic *Cmmm* Nb_2_O_5_ structure (*a* = 6.62 Å; *b* = 3.60 Å, *c* = 3.91 Å). The magnified [010] HAADF-STEM and ABF-STEM images together with the overlaid structural model are given as an inset in (a) (Nb atoms, orange spheres; O atoms, blue spheres).

High-resolution HAADF-STEM and simultaneously acquired ABF-STEM images along the main crystallographic zones, [010] and [110], agree with the structural model determined by PXRD (see inset in Figure 6). Using two complementary techniques, such as high-resolution HAADF-STEM and ABF-STEM, provides information about position of heavy (Nb, Ce) and light (O) elements. Regardless of the imperfect orientation of nanostructures due to their small size exactly along the zone axis, the overlaid structural model shows good correspondence to the experimental images. Due to the relatively large difference in atomic numbers between Nb(41) and Ce(58), it should be possible to distinguish positions of these two atoms in high-resolution HAADF-STEM images. However, no difference in atomic columns contrast in HAADF-STEM images was detected, suggesting very high dispersion of ceria species on the niobia surface.

**Figure 7:**
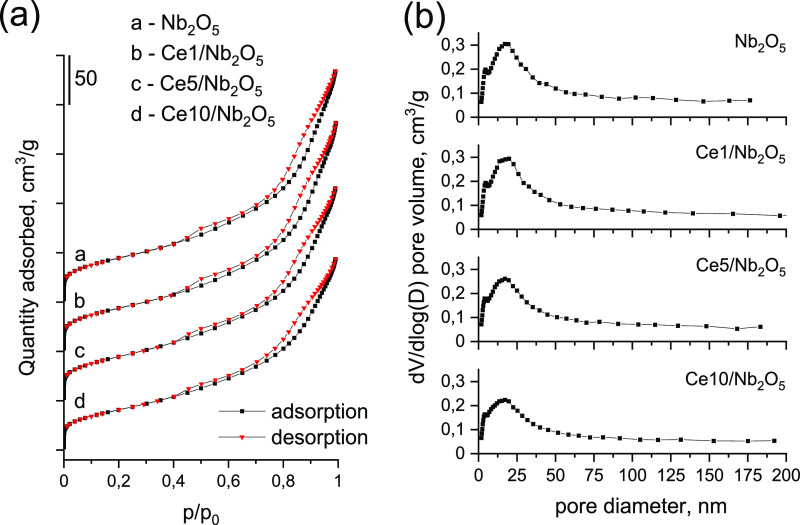
(a) Low-temperature nitrogen adsorption–desorption isotherms of catalysts. (b) Pore size distribution estimated for the catalysts from the adsorption branch of N_2_ isotherms using the BJH method.

Texture of the catalysts was characterized with the use of low-temperature nitrogen adsorption–desorption measurements. As can be seen from Figure 7a, all isotherms were of type IV(a), indicating the mesoporous structure of the catalysts.^43^ Textural parameters of materials estimated from nitrogen physisorption are summarized in Table 1. The highest BET surface area of 157 m^2^/g was observed for parent niobium pentoxide. Deposition of ceria species on niobia by wet impregnation led to a decrease in the catalyst surface area, but differences between samples were insufficient to justify the drastic difference in catalytic activities. The lowest surface area of 143 m^2^/g was characteristic of the catalyst containing the highest amount of ceria modifier, i.e., Ce10/Nb_2_O_5_. Figure 7b shows that loading of ceria on the niobia support also had a negligible influence on textural properties of the Nb_2_O_5_-based samples. All the catalysts exhibited a broad pore size distribution ranging from ca. 2 nm to more than 50 nm. The average pore size estimated for all the catalysts from adsorption branch using Barrett–Joyner–Halenda (BJH) method ranged from 8 to 9 nm (see Table 1). The negligible influence of ceria modifier on the BET surface area and pore size distribution estimated for the catalysts is further evidence of the high dispersion of ceria species on the niobia surface, which is also coherent with the presence of ceria hydroxyls in the IR spectra reported above.

**Figure 8:**
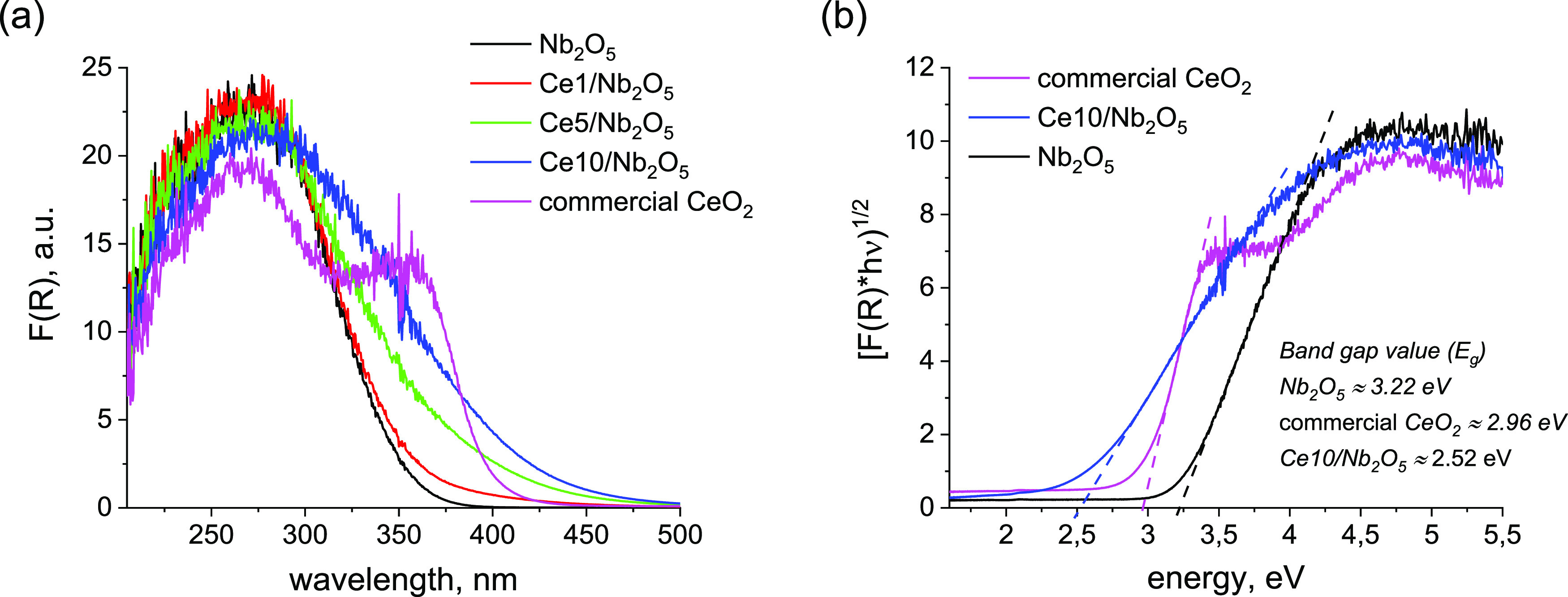
(a) Diffuse-reflectance UV–vis spectra of catalysts. (b) Results of band gap estimation using the Tauc plot method for selected catalysts.

The optical property of each catalyst was studied by diffuse-reflectance UV–vis spectroscopy. As can be seen from Figure 8a, parent niobia exhibited a broad absorption band with maximum absorbance at ca. 272 nm. According to the literature,^44^ this absorption band is typical of charge transfer transitions from O^2–^ to Nb^5+^, which are associated to the energy gap between the O 2p valence band and the Nb 4d conduction band of Nb_2_O_5_ bulk. Commercial CeO_2_ exhibited two absorption bands with maximum intensities centered at ca. 352 and 268 nm. According to the literature,^45,46^ these absorption bands are attributed to O^2–^ → Ce^4+^ charge transfer transitions and inter-band transitions, respectively. As can be seen from Figure 8a, deposition of ceria species on niobia increased the ability of the composite materials to absorb light in the range of wavelengths typical of O^2–^ → Ce^4+^ charge transfer transitions. It is worth noting that the increase in light absorption observed for ceria-modified samples was proportional to the amount of Ce. The higher concentration of ceria species in the composite material, the higher the materials ability to absorb light in the range of wavelengths typical of O^2–^ → Ce^4+^ charge transfer transitions.

Band gap values estimated for parent Nb_2_O_5_ and commercial CeO_2_ using Tauc’s plot method were 3.22 and 2.96 eV, respectively, and are in agreement with previous literature data concerning optical properties of Nb_2_O_5_^16,20,47^ and CeO_2_.^48,49^ Interestingly, the composite material prepared by a loading of 10 wt % of Ce on niobia by wet impregnation exhibited a significantly lower band gap value than that observed for both pristine metal oxides (see Figure 8b). Such a decrease in band gap value of the composite material may result from the formation of a large quantity of defect sites in the structure of CeO_2_ (e.g., Ce^4+^ ions with neighboring oxygen defects or Ce^3+^ ions), which shifted the absorption edge of ceria toward higher wavelengths (i.e., lower energy values).^50^

**Figure 9:**
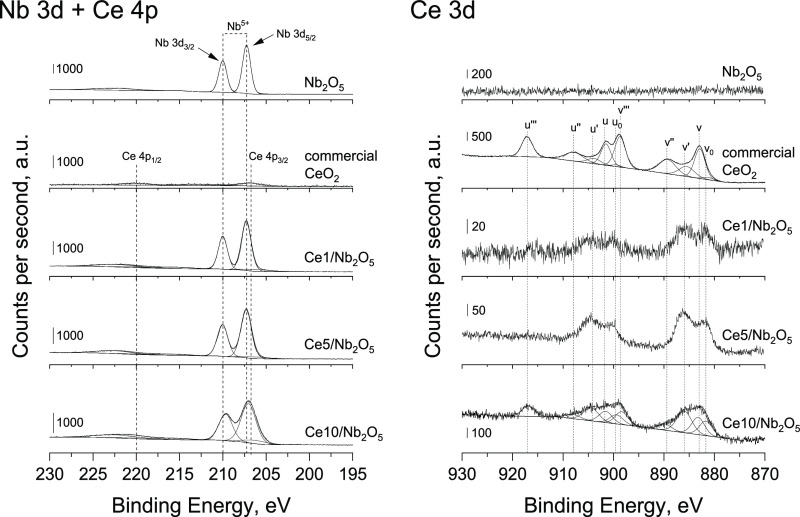
Nb 3d + Ce 4p (left) and Ce 3d (right) XP spectra of different catalysts studied in this work.

To get a deeper insight into the oxidation state of metals in the composite materials, the catalysts were characterized by X-ray photoelectron spectroscopy (Figure 9). The Nb 3d region of unmodified Nb_2_O_5_ was characterized by two components, namely, Nb 3d_5/2_ and Nb 3d_3/2_, with a binding energy of 207.3 and 210.0 eV, respectively. According to the literature,^51^ these components are assigned to Nb^5+^ species in bulk Nb_2_O_5_. In the case of ceria-modified samples prepared by wet impregnation, Nb 3d peaks overlapped with the peak typical of Ce 4p_3/2_. Precise deconvolution of experimental data led us to distinguish these components (see Figure 9). The only form of niobium species in all heterostructures was Nb^5+^. Deconvolution of Ce 3d spectra of commercial CeO_2_ allowed distinguishing 10 components, namely, v_0_, v, v′, v″, v‴, u_0_, u, u′, u″, and u‴ (see Figure 9). According to the literature,^52−54^ v, v″, v‴ and u, u″, u‴ are assigned to Ce^4+^ species, while v_0_, v′ and u_0_, u′ are attributed to Ce^3+^ species. Analysis of Ce 3d XP spectra of ceria-modified samples prepared by wet impregnation was more complex due to the low intensity of peaks at low ceria loadings. To avoid misinterpretation of experimental data, we have omitted deconvolution of Ce 3d spectra collected for Ce1/Nb_2_O_5_ and Ce5/Nb_2_O_5_. Nevertheless, it is important to stress that Ce 3d spectra recorded for these two samples had a different shape than that observed for commercial CeO_2_. The most important difference can be considered in terms of a very low relative contribution of components characteristic of Ce^4+^ species, in particular u‴, which is not overlapped with other peaks (see Figure 9).

More information about the oxidation state of cerium species in ceria-modified samples was provided by detailed analysis of Ce 3d spectra of Ce10/Nb_2_O_5_, for which the signal was the most intense. As shown in Figure 9, relative contribution of XP peaks characteristic of Ce^3+^ species was significantly more pronounced for Ce10/Nb_2_O_5_ than that observed for commercial CeO_2_. Since FTIR measurements for both samples did not reveal any noticeable IR band typical of methanol molecules adsorbed on Ce^3+^ cations, we claimed that a higher relative contribution of XP peaks characteristic of Ce^3+^ species established for Ce10/Nb_2_O_5_ resulted most probably from a higher concentration of defect sites in the structure of this catalyst (i.e., Ce^4+^ ions surrounded by oxygen vacancies in which electrons are trapped). However, the presence of some Ce^3+^ species in all ceria-modified Nb_2_O_5_ catalysts cannot be totally excluded. As it was documented by Baldim et al.,^55^ the concentration of defect sites on the surface of CeO_2_ nanoparticles often increases with decreasing particle size. Since ceria species in all catalysts prepared by wet impregnation were highly dispersed on the niobia surface, we claim that a high concentration of the above-mentioned defect sites in the structure of these materials is very likely. It is worth noting that our hypothesis is in agreement with results of UV–vis studies, in which a significant shift of the absorption edge of Ce10/Nb_2_O_5_ toward lower energy values was observed (see Figure 8b).

**Figure 10:**
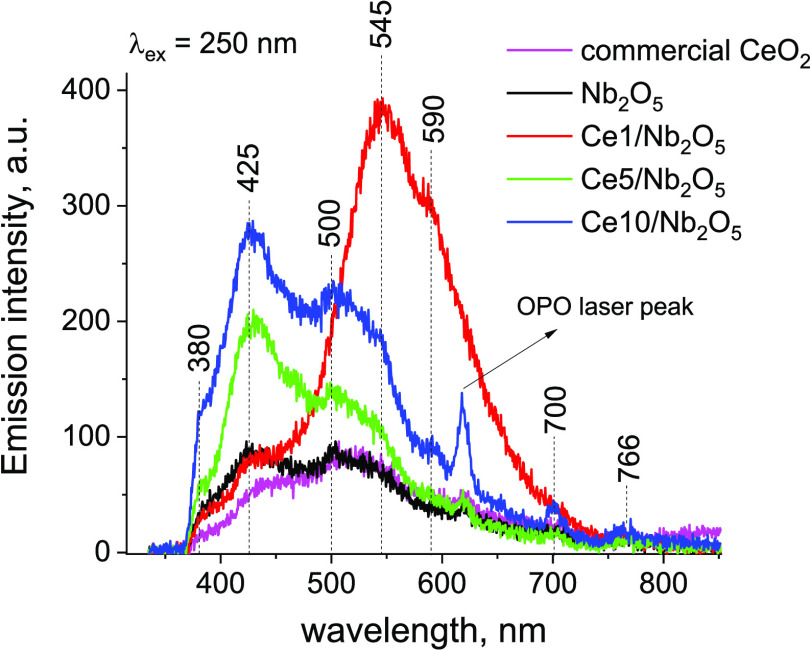
Photoluminescence spectra of catalysts.

To verify whether loading of ceria on niobia has any impact on recombination of charge carriers in the composite materials, we have performed photoluminescence measurements. According to the literature, photoluminescence emission mainly originated from radiative recombination of photo-generated electrons and holes trapped in the band tails of semiconductors.^56^ The higher the emission intensity, the higher the efficiency of charge carrier recombination.^57^ Parent niobium pentoxide exhibited three broad emission peaks with a maximum intensity at ca. 380, 425, and 500 nm (Figure 10).

The emission peaks at ca. 380 and 425 nm exhibit energy close to the optical band gap of Nb_2_O_5_ (3.22 eV; see Figure 8b) and may be attributed to near-band gap emission from Nb_2_O_5_ (recombination of photo-excited electrons in the Nb 4d conduction band and holes in the in the O 2p valance band of Nb_2_O_5_^47^), while the green emission at ca. 500 nm may be related to the presence of structure defects, such as distorted NbO_6_ octahedral groups^47,58^ (charge transfer transitions form the Nb^5+^ 4d^0^ state to O 2p orbital of oxygen ions in distorted NbO_6_ octahedral^59^). In the case of commercial CeO_2_, two main broad emission peaks at similar wavelengths as that observed for Nb_2_O_5_ can be identified (the first peak at ca. 425 nm and the second one at ca. 500 nm). The energy of the former emission peak (∼2.92 eV) is close to the optical band gap of commercial CeO_2_ (2.96 eV; see Figure 8b). Thus, this emission peak can be attributed to the direct band to band recombination of charge carriers in CeO_2_ (recombination of photo-excited electrons in the Ce 4f conduction band and holes in the O 2p valance band of CeO_2_). According to the literature,^60,61^ the latter broad emission peak can be attributed to the transitions from electronic energy levels of defects (mainly oxygen vacancies) localized below the Ce 4f band of the CeO_2_ nanoparticles to the O 2p valence band of CeO_2_. In the Nb_2_O_5_-based samples, loading of ceria species on niobia led to a significant increase in emission intensity of composite materials at ca. 425, 500, and 545 nm (Figure 10). Increased emission at ca. 425 nm may results both from promoted recombination of photo-excited electrons in the Nb 4d conduction band and holes in the O 2p valence band of Nb_2_O_5_ and/or from promoted recombination of photo-generated charged carries in CeO_2_. For Ce5/Nb_2_O_5_ and Ce10/Nb_2_O_5_, the intensity of the emission at 425 nm increases with the increase in the ceria loading. However, the Ce1/Nb_2_O_5_ catalyst, characterized by the lowest ceria loading, exhibited almost the same intensity as unmodified Nb_2_O_5_ and CeO_2_. Therefore, we hypothesize that the more pronounced emission at 425 nm observed for ceria-modified niobia catalysts resulted from a direct band to band recombination of charge carriers in CeO_2_ (recombination of photo-excited electrons in the Ce 4f conduction band and holes in the O 2p valance band of CeO_2_). The emission energy of the peaks at ca. 500 nm (2.48 eV) and 545 nm (2.28 eV) is similar to the optical band gap of Ce10/Nb_2_O_5_ (optical band gap resulted from the presence of CeO_2_ species with a high concentration of defect sites; *E*_g_ ≈ 2.52 eV; Figure 8b). Thus, the increase in intensity of these emission peaks observed for ceria-modified samples may be attributed to improved charge recombination related to transitions from electronic energy levels of defects (mainly oxygen vacancies) localized below the Ce 4f band of the CeO_2_ nanoparticles to the O 2p valence band of CeO_2_. As shown in Figure 10, the Ce1/Nb_2_O_5_ catalyst exhibited a significantly higher emission intensity at 500 and 545 nm than unmodified CeO_2_ and other niobia-based samples. We hypothesize that this phenomenon may result from differences in ceria loading. As documented by Baldim et al.,^55^ the concentration of defect sites on the surface of CeO_2_ nanoparticles often increases with decreasing particle size. One can expect that, for the Ce1/Nb_2_O_5_ sample, characterized by the lowest ceria loading, dispersion of ceria species and concentration of surface defects should be the highest from among all niobia-based catalysts. Thus, it is very likely that these two features of the Ce1/Nb_2_O_5_ catalyst may facilitate efficient recombination of the charge carries resulting from presence of ceria (charge recombination related to transitions from electronic energy levels of defects localized below the Ce 4f band of the CeO_2_ nanoparticles to the O 2p valence band of CeO_2_). This hypothesis is in agreement with results shown in Figure 10, in which emission related to the presence of defect sites was the most pronounced for the sample with the lowest ceria loading (i.e., Ce1/Nb_2_O_5_), while emission related to direct band to band recombination of the charge carries was the most intense for the samples with the highest concentration of ceria modifier loaded on the niobia surface (i.e., Ce10/Nb_2_O_5_). As far as differences in recombination of charge carriers are concerned, it is worth noting that improved emission of the Ce1/Nb_2_O_5_ catalyst at 545 nm may result not only from the presence of oxygen vacancies but also Ce^3+^ ions where the broad emission can be expected from 400 to 650 nm, depending on the host compound. Similar emission spectra were recorded for Y_3_Al_2_Ga_3_O_12_:Ce^3+^ ceramics and Sr_3_AlO_4_F:Ce^3+^ phosphors, where the maximum of Ce^3+^ emission occurred at around 500–550 nm due to the 5d^1^-^2^F_5/2_ and 5d^1^-^2^F_7/2_ transitions.^62−64^ As described in the XPS section, relative contribution of Ce 3d peaks typical of ceria defect sites (e.g., Ce^3+^ ions and/or Ce^4+^ ions surrounded by oxygen vacancies in which electrons are trapped; peaks labeled as u_0_, u′ and v_0_, v′ in Figure 9) was significantly higher for all ceria-modified Nb_2_O_5_ catalysts than that observed for commercial CeO_2_. Thus, the presence of some Ce^3+^ ions in niobia-based samples is very probable. The reason why the emission peak was less pronounced for materials containing a higher amount of ceria modifier (i.e., Ce5/Nb_2_O_5_ and Ce10/Nb_2_O_5_) can be related to concentration quenching between Ce^3+^ ions, which are highly sensitive for this phenomenon. Kolte et al. observed almost the total emission quenching of Sr_2_Al_2_SiO_7_:Ce^3+^ phosphors when the concentration of Ce^3+^ ion exceeded 2%.^65^ Therefore, it would be difficult to expect the emission of Ce^3+^ ions in Ce5/Nb_2_O_5_ and Ce10/Nb_2_O_5_ samples.

### 3.3 Discussion on the Role of Ceria in Deactivation of CeO_2_/Nb_2_O_5_ Heterostructures

**Figure 11:**
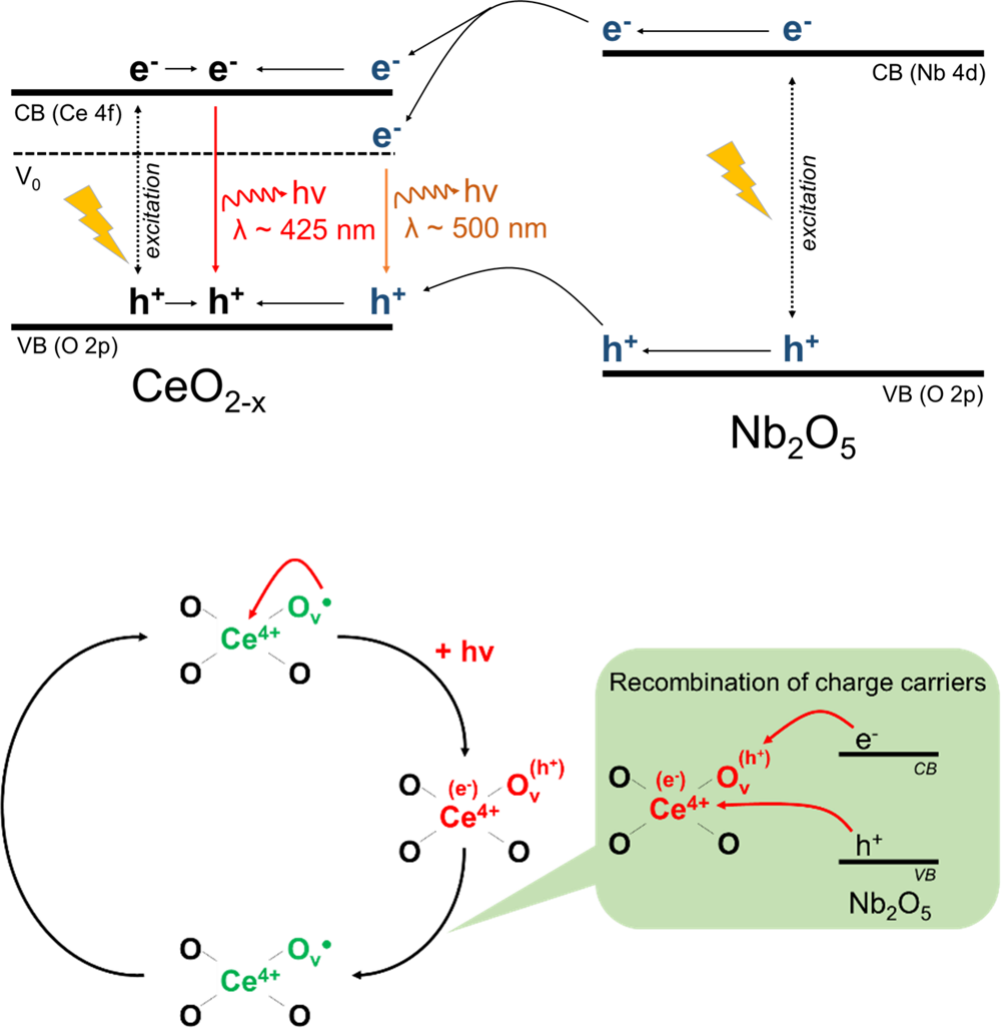
Schematic representation of charge transfer process resulting in deactivation of ceria-modified Nb_2_O_5_ samples prepared by wet impregnation; CB: conduction band, VB: valence band, V_0_: energy level of defects (mainly oxygen vacancies, O_V_) localized below the Ce 4f band of highly deficient ceria species (CeO_2–*x*_) loaded on Nb_2_O_5_.

Detailed characterization of as-prepared catalysts led us to observe that ceria species loaded on the niobia surface by wet impregnation were not only highly dispersed but also strongly interacted with the niobia support. As implied by UV–vis and XPS studies, such highly dispersed ceria species are substantially different from bulk CeO_2_. The heterostructures prepared by wet impregnation exhibited a much higher relative concentration of ceria lattice defects, which were responsible for changes in electronic and optical properties of ceria species. As revealed by photoluminescence studies, defect sites in the ceria lattice not only modified electronic and optical properties of ceria but they acted also as recombination centers for photo-excited electrons and holes. To shed more light on recombination of the photo-induced charge carries in CeO_2_/Nb_2_O_5_ heterostructures, we have predicted theoretically the conduction (*E*_CB_) and valence (*E*_VB_) band edges of pristine Nb_2_O_5_ and commercial CeO_2_ using the following empirical equations:^20^
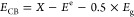
1
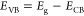
2where *X* is the absolute electronegativity of the semiconductor, obtained from the geometric mean of the electronegativity of its constituent atoms (*X* values for Nb_2_O_5_ and commercial CeO_2_ are 5.55^20^ and 5.57 eV,^49^ respectively); *E*_g_ is the band gap of the semiconductor (3.22 and 2.96 eV for Nb_2_O_5_ and commercial CeO_2_, respectively); *E*^e^ is the energy of free electrons *vs* the hydrogen scale (4.5 eV). The calculated conduction band edge potential of Nb_2_O_5_ was found to be −0.56 eV and is more negative than that of commercial CeO_2_ (−0.41 eV). The corresponding valence band edge potential estimated for Nb_2_O_5_ was 2.66 eV and more positive than that of commercial CeO_2_ (2.55 eV). We propose that irradiation of mechanical mixtures of the metal oxides with UV light resulted in the formation of photo-excited electrons (e^–^) and positively charged holes (h^+^) both in CeO_2_ and Nb_2_O_5_. According to the band edge positions, the as-formed h^+^ were transferred from the valence band of Nb_2_O_5_ to the valence band of commercial CeO_2_. At the same time, photo-excited electrons from the conduction band of Nb_2_O_5_ were transferred to conduction band of CeO_2_. Thus, the band edge alignments in CeO_2_/Nb_2_O_5_ heterostructures resulted in accumulation of both positively charged holes and photo-excited electrons in ceria species according to type I heterojunction.^66^ We hypothesize that such accumulation of the charge carriers in ceria promotes their efficient recombination and diminishes the photocatalytic performance of mechanical mixtures of the metal oxides. More pronounced deactivation of the heterostructures prepared by wet impregnation resulted from two unique features of these materials. First, ceria species in these materials were highly dispersed on the niobia surface, providing great interface between the modifier and the support and improving the photo-excited charge carriers transfer from one semiconductor to another. Second, such highly dispersed ceria species exhibited a high concentration of defect sites, which played the role of recombination centers for photo-generated electrons and holes, promoting their efficient recombination (see Figure 11).

As far as mechanism of the charge carrier separation is concerned, it is important to stress that formation of type II heterojunction is impossible in our case because of alignments of valence and conduction band edges in Nb_2_O_5_ and CeO_2_,^66^ even when we consider band edges estimated for highly deficient CeO_2–*x*_ species loaded on the niobia surface (see Figure 11). As far as other alternative mechanisms of charge carrier separation are concerned, one can expect that this process could proceed according to the Z-scheme mechanism.^66^ However, it is important to stress that this mechanism should lead to improved separation of the photo-excited charge carriers, and thus it should not result in total deactivation of the heterostructures. We can consider two possibilities. In the first scenario, photo-excited electrons from the conduction band of CeO_2_ could recombine with photo-generated holes localized in the valence band of Nb_2_O_5_. This process would lead to accumulation of photo-generated electrons in the conduction band of Nb_2_O_5_ and photo-generated holes in the valence band of CeO_2_. Since ceria itself is active in methanol photooxidation and Z-scheme mechanism would improve separation of the charge carriers, methanol molecules adsorbed on the surface of the composite catalysts should be efficiently oxidized by photo-generated holes localized in the valence band of CeO_2_. Thus, total deactivation of CeO_2_/Nb_2_O_5_ heterostructures cannot be explained by this mechanism of the charge separation. In the second scenario, photo-excited electrons from the conduction band of Nb_2_O_5_ could recombine with photo-generated holes localized in the valence band of CeO_2_. This process would lead to accumulation of photo-generated electrons in the conduction band of CeO_2_ and photo-generated holes in the valence band of Nb_2_O_5_. Since niobia is highly active in methanol photooxidation and Z-scheme mechanism would improve separation of the charge carriers, this mechanism should enhance the activity of CeO_2_/Nb_2_O_5_ heterostructures (it was not observed in our case). Thus, in a view of the above discussion, one can conclude that the formation of type I heterojunction is the most probable phenomenon that can fully explain more efficient recombination of the charge carriers in CeO_2_/Nb_2_O_5_ heterostructures and total deactivation of ceria-modified samples during methanol photooxidation.

## 4 Conclusions

We demonstrated that addition of ceria modifier to niobium pentoxide led to a significant decrease in the photocatalytic activity of CeO_2_/Nb_2_O_5_ nanostructures in methanol oxidation. We have established that, at any given concentration of ceria species on the niobia surface, the photocatalytic activity of both CeO_2_ and Nb_2_O_5_ in the composite material was totally quenched. *Operando*-IR studies demonstrated that deactivation of ceria-loaded Nb_2_O_5_ samples resulted from quenching the catalyst ability to oxidize adsorbed methanol molecules. Deeper insight into the mechanism of the photocatalytic process provided by photoluminescence measurements showed that deactivation of heterostructures and ineffective oxidation originated from promoting the recombination of photo-generated charge carriers. The main factors responsible for efficient recombination of photo-excited electrons and holes in the composite catalysts were high dispersion of ceria species on the niobia surface, which provided excessive interface between these two semiconductors, and high concentration of defect sites in the structure of such ultrafine ceria species (also shown by IR spectra of chemisorbed methanol), which acted as recombination centers for photo-excited charge carriers.

The results obtained in this study provide deep insight into the role of ceria modifier in controlling the photocatalytic activity of semiconductor-based photocatalysts. It is expected that this new fundamental knowledge about quenching of niobia activity by deposition of ceria species may play an important role in the development of new composite nanomaterials as UV filters. Furthermore, the knowledge about the origin of poisoning effect of Nb_2_O_5_ by ceria species is important for developing future heterogeneous ceria-modified photocatalysts too. In the case of the Nb_2_O_5_, modification with ceria will require a linker promoting a Z-scheme charge transfer (electron or hole) from niobia to ceria and minimizing the ceria/niobia interfaces.

## Supporting Information Available

FTIR spectra of catalyst surfaces at the beginning of methanol adsorption under dark conditions (Figure S1); crystal structure of the derived Nb_2_O_5_ model (*Cmmm*) (Figure S2); PXRD patterns for Ce/Nb_2_O_5_ samples collected with the Si (NIST Si 640d) internal standard (Figure S3); and bright-field and HAADF-STEM low magnification images of the Nb_2_O_5_ sample and overview bright-field HRTEM image and the corresponding ED pattern indexed based on the orthorhombic *Cmmm* structure (Figure S4) (PDF)

## References

[ref1] Montini T.; Melchionna M.; Monai M.; Fornasiero P. Fundamentals and Catalytic Applications of CeO_2_-Based Materials. Chem. Rev. 2016, 116, 5987–6041. 10.1021/acs.chemrev.5b00603. 27120134

[ref2] Bakkiyaraj R.; Bharath G.; Hasini Ramsait K.; Abdel-Wahab A.; Alsharaeh E. H.; Chen S. M.; Balakrishnan M. Solution Combustion Synthesis and Physico-Chemical Properties of Ultrafine CeO_2_ Nanoparticles and Their Photocatalytic Activity. RSC Adv. 2016, 6, 51238–51245. 10.1039/c6ra00382f.

[ref3] Cui Z.; Zhou H.; Wang G.; Zhang Y.; Zhang H.; Zhao H. Enhancement of the Visible-Light Photocatalytic Activity of CeO_2_ by Chemisorbed Oxygen in the Selective Oxidation of Benzyl Alcohol. New J. Chem. 2019, 43, 7355–7362. 10.1039/c9nj01098j.

[ref4] Majumder D.; Chakraborty I.; Mandal K.; Roy S. Facet-Dependent Photodegradation of Methylene Blue Using Pristine CeO_2_ Nanostructures. ACS Omega 2019, 4, 4243–4251. 10.1021/acsomega.8b03298. 31459631 PMC6648310

[ref5] Hamoud I. H.; Finqueneisel G.; Azambre B. Removal of Binary Dyes Mixtures with Opposite and Similar Charges by Adsorption, Coagulation/Flocculation and Catalytic Oxidation in the Presence of CeO_2_/H_2_O_2_ Fenton-like System. J. Environ. Manage. 2017, 195, 195–207. 10.1016/j.jenvman.2016.07.067. 27570146

[ref6] Cai W.; Chen F.; Shen X.; Chen L.; Zhang J. Enhanced Catalytic Degradation of AO7 in the CeO_2_-H_2_O_2_ system with Fe^3+^ doping. Appl. Catal., B 2010, 101, 160–168. 10.1016/j.apcatb.2010.09.031.

[ref7] Chen F.; Shen X.; Wang Y.; Zhang J. CeO_2_/H_2_O_2_ system Catalytic Oxidation Mechanism Study via a Kinetics Investigation to the Degradation of Acid Orange 7. Appl. Catal., B 2012, 121-122, 223–229. 10.1016/j.apcatb.2012.04.014.

[ref8] Zhu L.; Li H.; Xia P.; Liu Z.; Xiong D. Hierarchical ZnO Decorated with CeO_2_ Nanoparticles as the Direct Z-Scheme Heterojunction for Enhanced Photocatalytic Activity. ACS Appl. Mater. Interfaces 2018, 10, 39679–39687. 10.1021/acsami.8b13782. 30365889

[ref9] Muñoz-Batista M. J.; Gómez-Cerezo M. N.; Kubacka A.; Tudela D.; Fernández-García M. Role of Interface Contact in CeO_2_-TiO_2_ Photocatalytic Composite Materials. ACS Catal. 2014, 4, 63–72. 10.1021/cs400878b.

[ref10] Eskandarloo H.; Badiei A.; Behnajady M. A. TiO_2_/CeO_2_ Hybrid Photocatalyst with Enhanced Photocatalytic Activity: Optimization of Synthesis Variables. Ind. Eng. Chem. Res. 2014, 53, 7847–7855. 10.1021/ie403460d.

[ref11] Morlando A.; Chaki Borrás M.; Rehman Y.; Bakand S.; Barker P.; Sluyter R.; Konstantinov K. Development of CeO_2_ Nanodot Encrusted TiO_2_ Nanoparticles with Reduced Photocatalytic Activity and Increased Biocompatibility towards a Human Keratinocyte Cell Line. J. Mater. Chem. B 2020, 8, 4016–4028. 10.1039/d0tb00629g. 32347289

[ref12] Mueen R.; Morlando A.; Qutaish H.; Lerch M.; Cheng Z.; Konstantinov K. ZnO/CeO_2_ Nanocomposite with Low Photocatalytic Activity as Efficient UV Filters. J. Mater. Sci. 2020, 55, 6834–6847. 10.1007/s10853-020-04493-x.

[ref13] Su K.; Liu H.; Gao Z.; Fornasiero P.; Wang F. Nb_2_O_5_-Based Photocatalysts. Adv. Sci. 2021, 8, 2003156 10.1002/advs.202003156. PMC8061393 33898172

[ref14] Riente P.; Noël T. Application of Metal Oxide Semiconductors in Light-Driven Organic Transformations. Catal. Sci. Technol. 2019, 9, 5186–5232. 10.1039/c9cy01170f.

[ref15] Tamai K.; Hosokawa S.; Teramura K.; Shishido T.; Tanaka T. Synthesis of Niobium Oxide Nanoparticles with Plate Morphology Utilizing Solvothermal Reaction and Their Performances for Selective Photooxidation. Appl. Catal. B Environ. 2016, 182, 469–475. 10.1016/j.apcatb.2015.10.003.

[ref16] Kulkarni A. K.; Praveen C. S.; Sethi Y. A.; Panmand R. P.; Arbuj S. S.; Naik S. D.; Ghule A. V.; Kale B. B. Nanostructured N-Doped Orthorhombic Nb_2_O_5_ as an Efficient Stable Photocatalyst for Hydrogen Generation under Visible Light. Dalton Trans. 2017, 46, 14859–14868. 10.1039/c7dt02611k. 29043333

[ref17] Zhao Y.; Eley C.; Hu J.; Foord J. S.; Ye L.; He H.; Tsang S. C. E. Shape-Dependent Acidity and Photocatalytic Activity of Nb_2_O_5_ Nanocrystals with an Active TT (001) Surface. Angew. Chem., Int. Ed. Engl. 2012, 51, 3846–3849. 10.1002/anie.201108580. 22298466

[ref18] Nowak I.; Ziolek M. Niobium Compounds: Preparation, Characterization, and Application in Heterogeneous Catalysis. Chem. Rev. 1999, 99, 3603–3624. 10.1021/cr9800208. 11849031

[ref19] Ftouni K.; Lakiss L.; Thomas S.; Daturi M.; Fernandez C.; Bazin P.; El Fallah J.; El-Roz M. TiO_2_/Zeolite Bifunctional (Photo)Catalysts for a Selective Conversion of Methanol to Dimethoxymethane: On the Role of Brønsted Acidity. J. Phys. Chem. C 2018, 122, 29359–29367. 10.1021/acs.jpcc.8b10092.

[ref20] Yan J.; Wu G.; Guan N.; Li L. Nb_2_O_5_/TiO_2_ Heterojunctions: Synthesis Strategy and Photocatalytic Activity. Appl. Catal., B 2014, 152-153, 280–288. 10.1016/j.apcatb.2014.01.049.

[ref21] De Andrade F. V.; De Lima G. M.; Augusti R.; Coelho M. G.; Assis Y. P. Q.; Machado I. R. M. A New Material Consisting of TiO_2_ Supported on Nb_2_O_5_ as Photocatalyst for the Degradation of Organic Contaminants in Aqueous Medium. J. Environ. Chem. Eng. 2014, 2, 2352–2358. 10.1016/j.jece.2014.02.004.

[ref22] Lam S. M.; Sin J. C.; Satoshi I.; Abdullah A. Z.; Mohamed A. R. Enhanced Sunlight Photocatalytic Performance over Nb_2_O_5_/ZnO Nanorod Composites and the Mechanism Study. Appl. Catal. A Gen. 2014, 471, 126–135. 10.1016/j.apcata.2013.12.001.

[ref23] Wu J.; Li J.; Liu J.; Bai J.; Yang L. A Novel Nb_2_O_5_/Bi_2_WO_6_ Heterojunction Photocatalytic Oxidative Desulfurization Catalyst with High Visible Light-Induced Photocatalytic Activity. RSC Adv. 2017, 7, 51046–51054. 10.1039/c7ra09829d.

[ref24] Hashemzadeh F.; Gaffarinejad A.; Rahimi R. Porous p-NiO/n-Nb_2_O_5_ Nanocomposites Prepared by an EISA Route with Enhanced Photocatalytic Activity in Simultaneous Cr(VI) Reduction and Methyl Orange Decolorization under Visible Light Irradiation. J. Hazard. Mater. 2015, 286, 64–74. 10.1016/j.jhazmat.2014.12.038. 25557940

[ref25] Hong Y.; Li C.; Zhang G.; Meng Y.; Yin B.; Zhao Y.; Shi W. Efficient and Stable Nb_2_O_5_ Modified g-C_3_N_4_ Photocatalyst for Removal of Antibiotic Pollutant. Chem. Eng. J. 2016, 299, 74–84. 10.1016/j.cej.2016.04.092.

[ref26] Ferraz N. P.; Nogueira A. E.; Marcos F. C. F.; Machado V. A.; Rocca R. R.; Assaf E. M.; Asencios Y. J. O. CeO_2_–Nb_2_O_5_ Photocatalysts for Degradation of Organic Pollutants in Water. Rare Met. 2020, 39, 230–240. 10.1007/s12598-019-01282-7.

[ref27] Tatibouet J. M. Methanol Oxidation as a Catalytic Surface Probe. Appl. Catal., A 1997, 148, 213–252. 10.1016/S0926-860X(96)00236-0.

[ref28] Kähler K.; Holz M. C.; Rohe M.; Van Veen A. C.; Muhler M. Methanol Oxidation as Probe Reaction for Active Sites in Au/ZnO and Au/TiO_2_ Catalysts. J. Catal. 2013, 299, 162–170. 10.1016/j.jcat.2012.12.001.

[ref29] Crampton A. S.; Cai L.; Janvelyan N.; Zheng X.; Friend C. M. Methanol Photo-Oxidation on Rutile TiO_2_ Nanowires: Probing Reaction Pathways on Complex Materials. J. Phys. Chem. C 2017, 121, 9910–9919. 10.1021/acs.jpcc.7b01385.

[ref30] Murayama T.; Chen J.; Hirata J.; Matsumoto K.; Ueda W. Hydrothermal Synthesis of Octahedra-Based Layered Niobium Oxide and Its Catalytic Activity as a Solid Acid. Catal. Sci. Technol. 2014, 4, 4250–4257. 10.1039/C4CY00713A.

[ref31] Toby B. H.; Von Dreele R. B. GSAS-II: The Genesis of a Modern Open-Source All Purpose Crystallography Software Package. J. Appl. Crystallogr. 2013, 46, 544–549. 10.1107/S0021889813003531.

[ref32] Wuttke S.; Bazin P.; Vimont A.; Serre C.; Seo Y. K.; Hwang Y. K.; Chang J. S.; Férey G.; Daturi M. Discovering the Active Sites for C3 Separation in MIL-100(Fe) by Using Operando IR Spectroscopy. Chem. - Eur. J. 2012, 18, 11959–11967. 10.1002/chem.201201006. 22890853

[ref33] El-Roz M.; Bazin P.; Thibault-Starzyk F. An Operando-IR Study of Photocatalytic Reaction of Methanol on New *BEA Supported TiO_2_ Catalyst. Catal. Today 2013, 205, 111–119. 10.1016/j.cattod.2012.08.023.

[ref34] El-Roz M.; Kus M.; Cool P.; Thibault-Starzyk F. New Operando IR Technique to Study the Photocatalytic Activity and Selectivity of TiO_2_ nanotubes in Air Purification: Influence of Temperature, UV Intensity, and VOC Concentration. J. Phys. Chem. C 2012, 116, 13252–13263. 10.1021/jp3034819.

[ref35] El-Roz M.; Bazin P.; Daturi M.; Thibault-Starzyk F. On the Mechanism of Methanol Photooxidation to Methylformate and Carbon Dioxide on TiO_2_: An Operando-FTIR Study. Phys. Chem. Chem. Phys. 2015, 17, 11277–11283. 10.1039/C5CP00726G. 25835980

[ref36] Binet C.; Daturi M.; Lavalley J.-C. IR Study of Polycrystalline Ceria Properties in Oxidised and Reduced States. Catal. Today 1999, 50, 207–225. 10.1016/S0920-5861(98)00504-5.

[ref37] Daturi M.; Finocchio E.; Binet C.; Lavalley J. C.; Fally F.; Perrichon V. Study of Bulk and Surface Reduction by Hydrogen of Ce*_x_*Zr_1-*x*_O_2_ Mixed Oxides Followed by FTIR Spectroscopy and Magnetic Balance. J. Phys. Chem. B 1999, 103, 4884–4891. 10.1021/jp9905981.

[ref38] Kähler K.; Holz M. C.; Rohe M.; Strunk J.; Muhler M. Probing the Reactivity of ZnO and Au/ZnO Nanoparticles by Methanol Adsorption: A TPD and DRIFTS Study. ChemPhysChem 2010, 11, 2521–2529. 10.1002/cphc.201000282. 20635374

[ref39] Daturi M.; Binet C.; Lavalley J.-C.; Galtayries A.; Sporken R. Surface Investigation on Ce_x_Zr_1-x_O_2_ Compounds. Phys. Chem. Chem. Phys. 1999, 1, 5717–5724. 10.1039/a905758g.

[ref40] Kaichev V. V.; Popova G. Y.; Chesalov Y. A.; Saraev A. A.; Zemlyanov D. Y.; Beloshapkin S. A.; Knop-Gericke A.; Schlögl R.; Andrushkevich T. V.; Bukhtiyarov V. I. Selective Oxidation of Methanol to Form Dimethoxymethane and Methyl Formate over a Monolayer V_2_O_5_/TiO_2_ Catalyst. J. Catal. 2014, 311, 59–70. 10.1016/j.jcat.2013.10.026.

[ref41] Moulin B.; Oliviero L.; Bazin P.; Daturi M.; Costentin G.; Maugé F. How to Determine IR Molar Absorption Coefficients of Co-Adsorbed Species? Application to Methanol Adsorption for Quantification of MgO Basic Sites. Phys. Chem. Chem. Phys. 2011, 13, 10797–10807. 10.1039/c0cp02767g. 21552623

[ref42] Schmid S.; Fung V. Incommensurate Modulated Structures in the Ta_2_O_5_-Al_2_O_3_ System. Aust. J. Chem. 2012, 65, 851–859. 10.1071/CH12080.

[ref43] Thommes M.; Kaneko K.; Neimark A. V.; Olivier J. P.; Rodriguez-Reinoso F.; Rouquerol J.; Sing K. S. W. Physisorption of Gases, with Special Reference to the Evaluation of Surface Area and Pore Size Distribution (IUPAC Technical Report). Pure Appl. Chem. 2015, 87, 1051–1069. 10.1515/pac-2014-1117.

[ref44] Armaroli T.; Busca G.; Carlini C.; Giuttari M.; Raspolli Galletti A. M.; Sbrana G. Acid Sites Characterization of Niobium Phosphate Catalysts and Their Activity in Fructose Dehydration to 5-Hydroxymethyl-2-Furaldehyde. J. Mol. Catal. A: Chem. 2000, 151, 233–243. 10.1016/S1381-1169(99)00248-4.

[ref45] Liu L.; Yao Z.; Liu B.; Dong L. Correlation of Structural Characteristics with Catalytic Performance of CuO/Ce_x_Zr_1-x_O_2_ catalysts for NO Reduction by CO. J. Catal. 2010, 275, 45–60. 10.1016/j.jcat.2010.07.024.

[ref46] Reddy B. M.; Bharali P.; Saikia P.; Park S. E.; Van Den Berg M. W. E.; Muhler M.; Grünert W. Structural Characterization and Catalytic Activity of Nanosized Ce_x_M_1-x_O_2_ (M = Zr and Hf) Mixed Oxides. J. Phys. Chem. C 2008, 112, 11729–11737. 10.1021/jp802674m.

[ref47] Zhou Y.; Qiu Z.; Lü M.; Zhang A.; Ma Q. Preparation and Spectroscopic Properties of Nb_2_O_5_ Nanorods. J. Lumin. 2008, 128, 1369–1372. 10.1016/j.jlumin.2008.01.001.

[ref48] Channei D.; Inceesungvorn B.; Wetchakun N.; Ukritnukun S.; Nattestad A.; Chen J.; Phanichphant S. Photocatalytic Degradation of Methyl Orange by CeO_2_ and Fe–doped CeO_2_ Films under Visible Light Irradiation. Sci. Rep. 2015, 4, 5757 10.1038/srep05757. PMC5385822 25169653

[ref49] Arul N. S.; Mangalaraj D.; Ramachandran R.; Grace A. N.; Han J. I. Fabrication of CeO_2_/Fe_2_O_3_ Composite Nanospindles for Enhanced Visible Light Driven Photocatalysts and Supercapacitor Electrodes. J. Mater. Chem. A 2015, 3, 15248–15258. 10.1039/c5ta02630j.

[ref50] Ansari S. A.; Khan M. M.; Ansari M. O.; Kalathil S.; Lee J.; Cho M. H. Band Gap Engineering of CeO_2_ Nanostructure Using an Electrochemically Active Biofilm for Visible Light Applications. RSC Adv. 2014, 4, 16782–16791. 10.1039/c4ra00861h.

[ref51] Liu H.; Gao N.; Liao M.; Fang X. Hexagonal-like Nb_2_O_5_ Nanoplates-Based Photodetectors and Photocatalyst with High Performances. Sci. Rep. 2015, 5, 7716 10.1038/srep07716. 25578788 PMC4648379

[ref52] Bêche E.; Charvin P.; Perarnau D.; Abanades S.; Flamant G. Ce 3d XPS Investigation of Cerium Oxides and Mixed Cerium Oxide (Ce_x_Ti_y_O_z_). Surf. Interface Anal. 2008, 40, 264–267. 10.1002/sia.2686.

[ref53] Pereira-Hernández X. I.; DeLaRiva A.; Muravev V.; Kunwar D.; Xiong H.; Sudduth B.; Engelhard M.; Kovarik L.; Hensen E. J. M.; Wang Y.; Datye A. K. Tuning Pt-CeO_2_ Interactions by High-Temperature Vapor-Phase Synthesis for Improved Reducibility of Lattice Oxygen. Nat. Commun. 2019, 10, 1358 10.1038/s41467-019-09308-5. 30911011 PMC6433950

[ref54] Lei W.; Zhang T.; Gu L.; Liu P.; Rodriguez J. A.; Liu G.; Liu M. Surface-Structure Sensitivity of CeO_2_ Nanocrystals in Photocatalysis and Enhancing the Reactivity with Nanogold. ACS Catal. 2015, 5, 4385–4393. 10.1021/acscatal.5b00620.

[ref55] Baldim V.; Bedioui F.; Mignet N.; Margaill I.; Berret J. F. The Enzyme-like Catalytic Activity of Cerium Oxide Nanoparticles and Its Dependency on Ce^3+^ Surface Area Concentration. Nanoscale 2018, 10, 6971–6980. 10.1039/c8nr00325d. 29610821

[ref56] Georgiev R.; Georgieva B.; Vasileva M.; Ivanov P.; Babeva T. Optical Properties of Sol-Gel Nb_2_O_5_ Films with Tunable Porosity for Sensing Applications. Adv. Condens. Matter Phys. 2015, 2015, 1–8. 10.1155/2015/403196.

[ref57] Khan M. E.; Khan M. M.; Cho M. H. Ce^3+^-Ion, Surface Oxygen Vacancy, and Visible Light-Induced Photocatalytic Dye Degradation and Photocapacitive Performance of CeO_2_-Graphene Nanostructures. Sci. Rep. 2017, 7, 5928 10.1038/s41598-017-06139-6. 28724968 PMC5517655

[ref58] Chen Q. Nb_2_O_5_ Improved Photoluminescence, Magnetic and Faraday Rotation Properties of Magneto-Optical Glasses. J. Non-Cryst. Solids 2019, 519, 119451 10.1016/j.jnoncrysol.2019.05.027.

[ref59] Zeng H.; Song J.; Chen D.; Yuan S.; Jiang X.; Cheng Y.; Yang Y.; Chen G. Three-Photon-Excited Upconversion Luminescence of Niobium Ions Doped Silicate Glass by a Femtosecond Laser Irradiation. Opt. Express 2008, 16, 6502 10.1364/oe.16.006502. 18545353

[ref60] Kumar S.; Ojha A. K.; Patrice D.; Yadav B. S.; Materny A. One-Step *in situ* Synthesis of CeO_2_ Nanoparticles Grown on Reduced Graphene Oxide as an Excellent Fluorescent and Photocatalyst Material under Sunlight Irradiation. Phys. Chem. Chem. Phys. 2016, 18, 11157–11167. 10.1039/c5cp04457j. 27049142

[ref61] Hemalatha K. S.; Rukmani K. Synthesis, Characterization and Optical Properties of Polyvinyl Alcohol-Cerium Oxide Nanocomposite Films. RSC Adv. 2016, 6, 74354–74366. 10.1039/c6ra11126b.

[ref62] Silveira L. G. D.; Cótica L. F.; Santos I. A.; Belançon M. P.; Rohling J. H.; Baesso M. L. Processing and Luminescence Properties of Ce:Y_3_Al_5_O_12_ and Eu:Y_3_Al_5_O_12_ Ceramics for White-Light Applications. Mater. Lett. 2012, 89, 86–89. 10.1016/j.matlet.2012.08.106.

[ref63] Santos J. C. A.; Silva E. P.; Sampaio D. V.; Silva D. C.; Souza N. R. S.; Kucera C.; Ballato J.; Silva R. S. Effect of the Ce^3+^ Concentration on Laser-Sintered YAG Ceramics for White LEDs Applications. J. Eur. Ceram. Soc. 2020, 40, 3673–3678. 10.1016/j.jeurceramsoc.2020.03.069.

[ref64] Boiko V.; Zeler J.; Markowska M.; Dai Z.; Gerus A.; Bolek P.; Zych E.; Hreniak D. Persistent Luminescence from Y_3_Al_2_Ga_3_O_12_ Doped with Ce^3+^ and Cr^3+^ after X-Ray and Blue Light Irradiation. J. Rare Earths 2019, 37, 1200–1205. 10.1016/j.jre.2019.03.010.

[ref65] Kolte M.; Pawade V. B.; Dhoble S. J. Quenching and Dipole–dipole Interactions in Sr_2_Al_2_SiO_7_:Ce^3+^ Host Lattice. Appl. Phys. A: Mater. Sci. Process. 2016, 122, 59 10.1007/s00339-015-9579-0.

[ref66] Low J.; Yu J.; Jaroniec M.; Wageh S.; Al-Ghamdi A. A. Heterojunction Photocatalysts. Adv. Mater. 2017, 29, 1601694 10.1002/adma.201601694. 28220969

