# Supplementary material for: Unraveling the Origin of Photocatalytic Deactivation in CeO2/Nb2O5 Heterostructure Systems during Methanol Oxidation: Insight into the Role of Cerium Species
Source: J Phys Chem C Nanomater Interfaces. 2021 Jun 2;125(23):12650–62. doi: 10.1021/acs.jpcc.1c02812 (PMC8279704; doi:10.1021/acs.jpcc.1c02812)
Supplement: Supplementary file 1 — jp1c02812_si_001.pdf [file jp1c02812_si_001.pdf]

# Supporting Information

*for The Journal of Physical Chemistry C*

## Unravelling the Origin of Photocatalytic Deactivation in $CeO_2/Nb_2O_5$ Heterostructure Systems during Methanol Oxidation - Insight into the Role of Cerium Species

Lukasz Wolski[a,b,*], Oleg I. Lebedev[c], Colin P. Harmer[d,e], Kirill Kovnir[d,e], Hanen Abdelli[b], Tomasz Grzyb[f], Marco Daturi[b], Mohamad El-Roz[b,*],

[a] *Faculty of Chemistry, Adam Mickiewicz University, Poznań, Uniwersytetu Poznańskiego 8, 61-614 Poznań, Poland*

[b] *Normandie Univ, ENSICAEN, UNICAEN, CNRS, Laboratoire Catalyse et Spectrochimie, 14050 Caen, France*

[c] *Normandie Univ, ENSICAEN, UNICAEN, CNRS, Laboratoire CRISMAT, 14050 Caen, France*

[d] *Iowa State University, Department of Chemistry, Ames, Iowa 50011, United States of America;*

[e] *U.S. Department of Energy, Ames Laboratory, Ames, Iowa, 50011, United States of America*

[f] *Department of Rare Earths, Faculty of Chemistry, Adam Mickiewicz University, Poznań, Uniwersytetu Poznańskiego 8, 61-614 Poznań, Poland*

** corresponding authors at:*

*Adam Mickiewicz University, Poznań, Poland; e-mail: wolski.lukasz@amu.edu.pl (L. Wolski)*

*Laboratoire Catalyse et Spectrochimie, France; e-mail: mohamad.elroz@ensicaen.fr (M. El-Roz)*

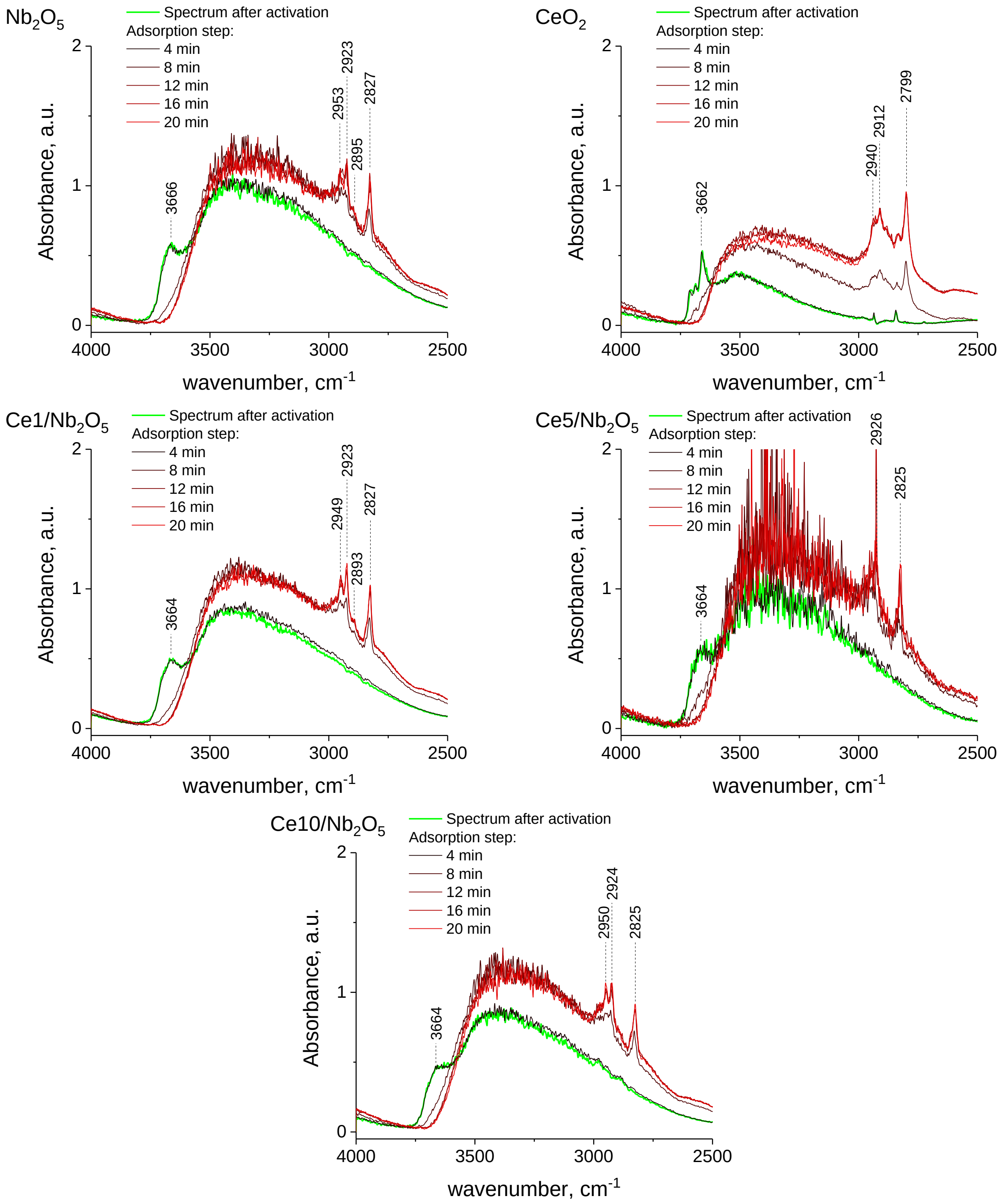


**Fig. S1.** FTIR spectra of catalyst surfaces at the beginning of methanol adsorption under dark conditions.

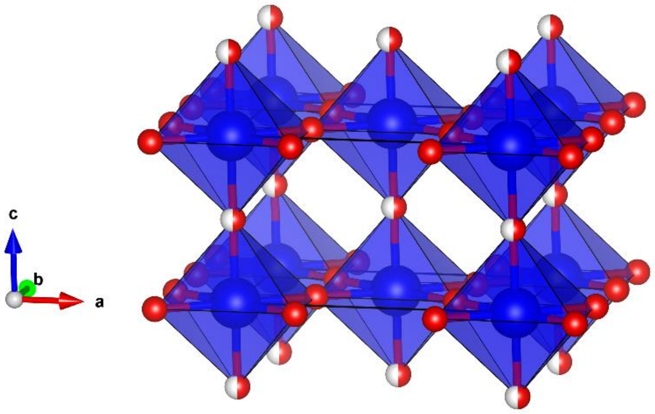


**Fig. S2**: Crystal structure of the derived $Nb_2O_5$ model (*Cmmm*). Niobium (blue spheres), oxygen (red spheres), Nb-O octahedra (blue). Partially occupied sites are shown as red/white spheres.

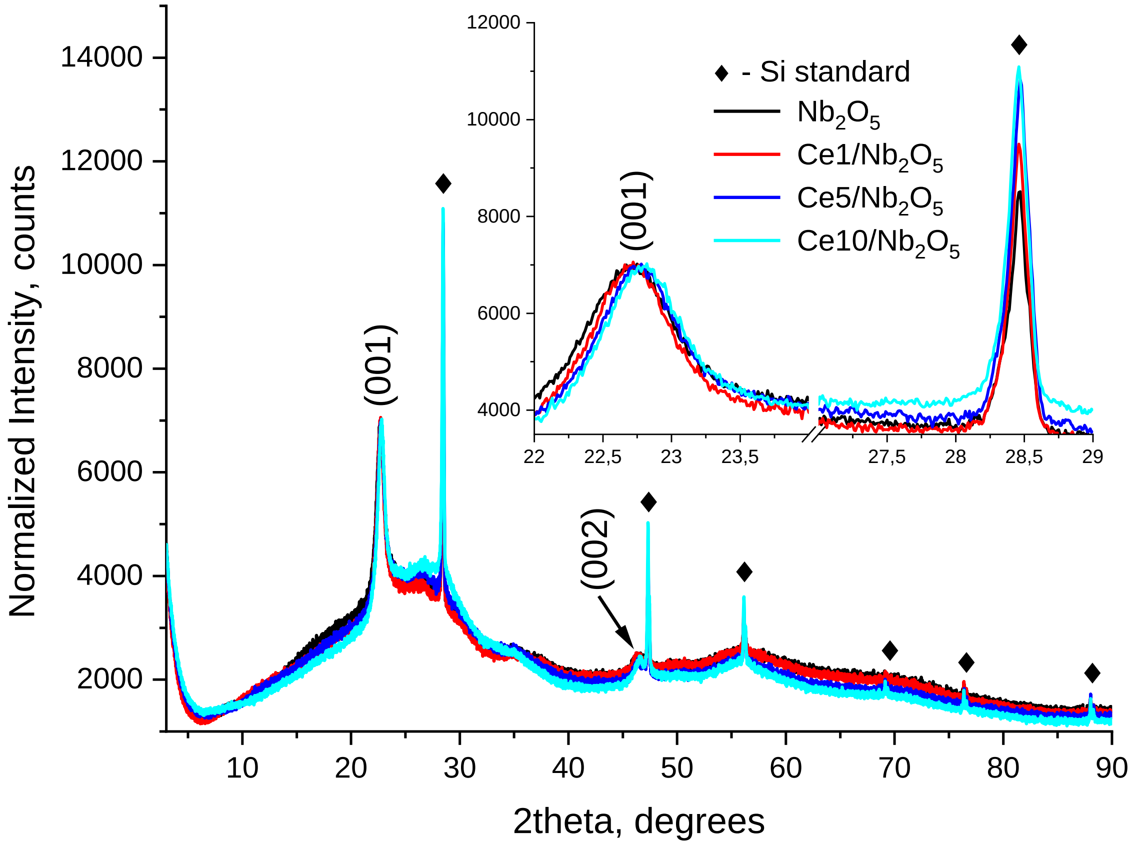


**Fig. S3**: PXRD patterns for Ce/$Nb_2O_5$ samples prepared by wet impregnation (Cu Kα, λ = 1.5406 Å) normalized to the intensity of (001) peak. Collected with Si (NIST Si 640d) internal standard.

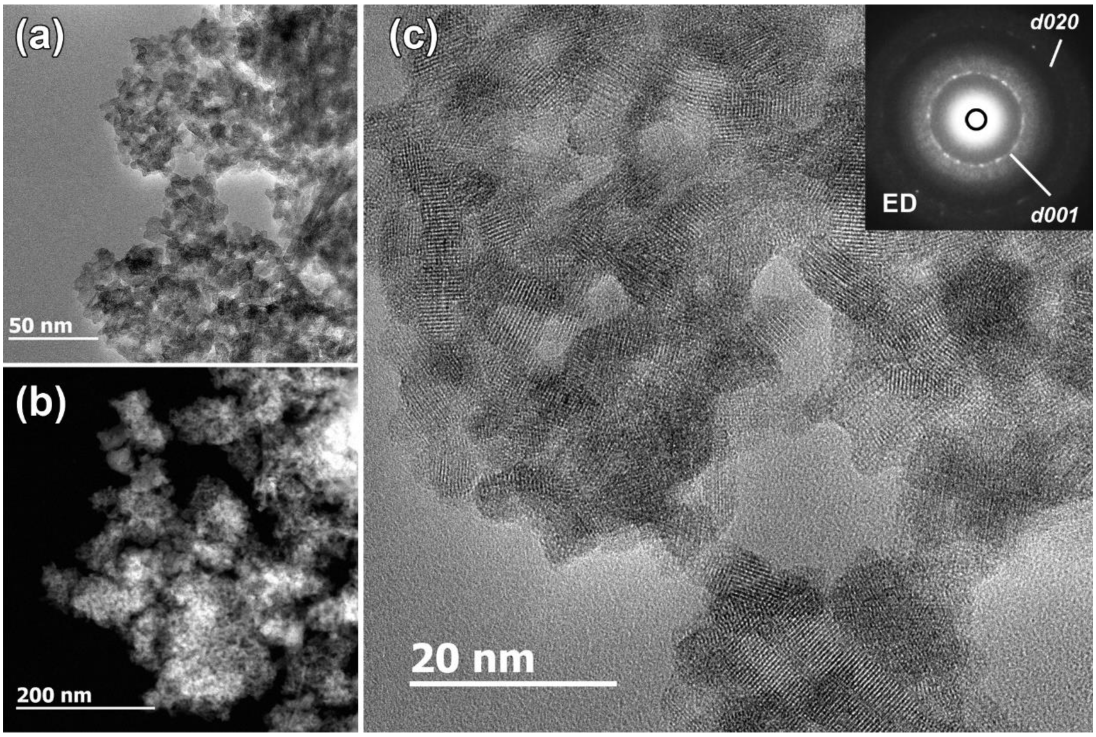


**Fig.S4 (a)** Bright field and **(b)** HAADF-STEM low magnification images of $Nb_2O_5$ sample; **(c)** overview bright field HRTEM image and corresponding ED pattern indexed based on orthorhombic *Cmmm* structure.
